# Tomato plants rather than fertilizers drive microbial community structure in horticultural growing media

**DOI:** 10.1038/s41598-019-45290-0

**Published:** 2019-07-02

**Authors:** Oliver Grunert, Ana A. Robles-Aguilar, Emma Hernandez-Sanabria, Silvia D. Schrey, Dirk Reheul, Marie-Christine Van Labeke, Siegfried E. Vlaeminck, Tom G. L. Vandekerckhove, Mohamed Mysara, Pieter Monsieurs, Vicky M. Temperton, Nico Boon, Nicolai D. Jablonowski

**Affiliations:** 1 Center for Microbial Ecology and Technology (CMET), Ghent University, Coupure Links 653, 9000 Gent, Belgium; 2 Greenyard, Skaldenstraat 7a, 9042 Desteldonk, Belgium; 3 Forschungszentrum Jülich GmbH, Institute of Bio- and Geosciences, IBG-2: Plant Sciences, 52428 Jülich, Germany; 4 Laboratory of Analytical Chemistry and Applied Ecochemistry, Faculty of Bioscience Engineering, Ghent University, Coupure Links 653, B-9000 Ghent, Belgium; 5 Department of Plant and Crops, Ghent University, Coupure Links 653, 9000 Gent, Belgium; 6 Research Group of Sustainable Energy, Air and Water Technology, Department of Bioscience Engineering, University of Antwerp, Groenenborgerlaan 171, 2020 Antwerpen, Belgium; 7 Unit of Microbiology, Belgian Nuclear Research Center (SCK•CEN), Mol, Belgium; 8 Department of Bioscience Engineering, Vrije Universiteit Brussel, Brussels, Belgium; 9 Unit Health, Flemish Institute for Technological Research (VITO), Mol, Belgium; 10 Institute of Ecology, Leuphana University Lüneburg, Universitätsallee 1, D-21335 Lüneburg, Germany

**Keywords:** Plant ecology, Plant physiology

## Abstract

Synthetic fertilizer production is associated with a high environmental footprint, as compounds typically dissolve rapidly leaching emissions to the atmosphere or surface waters. We tested two recovered nutrients with slower release patterns, as promising alternatives for synthetic fertilizers: struvite and a commercially available organic fertilizer. Using these fertilizers as nitrogen source, we conducted a rhizotron experiment to test their effect on plant performance and nutrient recovery in juvenile tomato plants. Plant performance was significantly improved when organic fertilizer was provided, promoting higher shoot biomass. Since the microbial community influences plant nitrogen availability, we characterized the root-associated microbial community structure and functionality. Analyses revealed distinct root microbial community structure when different fertilizers were supplied. However, plant presence significantly increased the similarity of the microbial community over time, regardless of fertilization. Additionally, the presence of the plant significantly reduced the potential ammonia oxidation rates, implying a possible role of the rhizosheath microbiome or nitrification inhibition by the plant. Our results indicate that nitrifying community members are impacted by the type of fertilizer used, while tomato plants influenced the potential ammonia-oxidizing activity of nitrogen-related rhizospheric microbial communities. These novel insights on interactions between recovered fertilizers, plant and associated microbes can contribute to develop sustainable crop production systems.

## Introduction

About 95% of greenhouse vegetables, and especially tomatoes in Europe, U.S.A., and Canada are produced in soil-less culture systems^1^ using artificial growing media that provide plants with necessary nutrients. Intensive horticulture, as well as intensive agriculture heavily rely on the input of inorganic and organic fertilizers to sustain food production^2^. The synthetic fertilizer production is associated with high environmental footprint. Therefore, it is meaningful to move towards a sustainable crop production via recovery/reuse of the nutrients in form of alternative fertilizers^3^. The heavy environmental impact of mineral fertilizers in traditional agriculture is aggravated by high nitrogen leaching, as compounds are typically rapidly dissolved^4^. Timing, ratio, and quantity of nutrients are fundamental for optimal plant growth, because the nutrient demand of the plant may not be concomitant with the release from the fertilizers^5,6^.

Tomatoes can take up nitrogen in the form of ammonium or nitrate^7^. The N contained in organic fertilizers is converted into NH_4_^+^ and NO_3_^−^ by microorganisms to be plant-available. However, plants are capable of taking other organic N sources like simple amino acids^7^. One major process in nitrogen cycling is the conversion of ammonium (NH_4_^+^) or ammonia (NH_3_) to nitrite (NO_2_^−^), which is called ‘ammonia oxidation’ or ‘nitritation’. The further transformation of nitrite (NO_2_^−^), into nitrate (NO_3_^−^), is called ‘nitrite-oxidation’ or ‘nitratation’. Ammonia-oxidizing archaea (AOA) and ammonia-oxidizing bacteria (AOB) oxidize ammonia to nitrite. AOA and AOB cohabit the vast majority of terrestrial ecosystems^8^, but they may occupy different ecological niches^9^. Nitrite-oxidizing bacteria (NOB) can utilize nitrite as energy source and carbon dioxide as the main carbon source^10^ and they convert nitrite (NO_2_^−^) to nitrate^11^.

The demand for organically grown products has increased during recent years^12^. Thus, organic fertilizers represent a sustainable alternative to mineral fertilizers and can be produced on-farm (e.g. slurries, poultry manures digestate) or off-farm (e.g. as residues from food industry)^13^. Additionally, recycled sources of inorganic nutrients are increasingly becoming available, recovering N from wastewater, sludge or manure through nitrification (e.g. stabilized urine)^14^, stripping/absorption (e.g. (NH_4_)_2_SO_4_)) or crystallization with magnesium and phosphorus, yielding struvite (MgNH_4_PO_4_.6H_2_O), which is applicable as slow-release fertilizer^15–17^.

**Figure 1:**
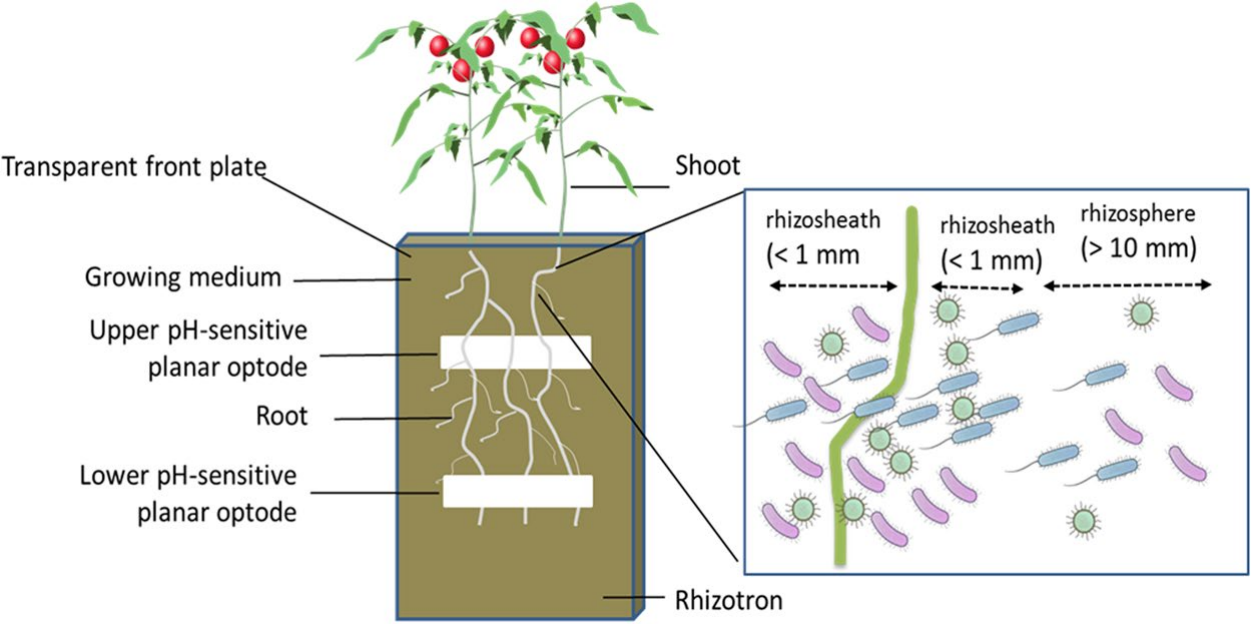
Rhizotron design indicating the removable side of the transparent polycarbonate plate that allows the visualization of the roots, and the location of the planar pH-optodes. The planar optode set-up consisted of a foil containing the sensor fixed to the transparent inner side of the rhizotron surface, which comes into contact with roots. The figure also indicates the places where substrate samples were taken at two distances to the root: rhizosphere at approx. 10 mm from the root and rhizosheath samples taken directly at the root (less than 1 mm distance to a root).

Little research has been conducted on how to efficiently apply recycled nutrients, and how plants and their environment react to them^18^. A key factor that might impact nutrient-use efficiency from these fertilizers in the organic growing media is the root-associated microbial community and its effects on nutrient mobilization, immobilization, and conversion^19^. Plants can impact not only the growing media physically adhered to the roots (rhizosheath), but the effect can reach up to the entire rhizosphere through root exudates^20^. Hence, microbial community structure and activity, nutrient cycling, pH and consequently plant growth will be impacted^21^. We defined rhizosheath (Fig. 1) as the soil that physically adheres to the roots system (in the range of less than 1 mm distant from a root), while rhizosphere refers to the soil influenced by the root (within a region of 1 cm distant from the root), as suggested by Pang *et al*.^20^. “Bulk” was defined as the growing medium within a rhizotron without tomato plants growing. Rhizosheath microbial communities during early plant development are different to those in the rhizosphere, indicating an active plant-shaping effect on the community at the beginning of the life cycle and a slowdown of this effect as plant ages^22^. Changes in the microbial community composition in the rhizosphere might be driven by the plant-microbe competition for nutrients in the growing medium, such as phosphorous and nitrogen^23,24^, or by the form of the nitrogen as ammonium or as nitrate^25^. Indeed nitrogen can change the abundance of ammonia oxidizing bacteria (AOB) and nitrite oxidizing bacteria (NOB) in the soils^26,27^ and shift their activities^26^. Many studies have analysed the impact of different N fertilizers on the microbial community. However, we focused on portraying a connection that is yet to be explored: how different ammonium sources (e.g. struvite and organic-N) and the associated nitrogen transformation processes influence the rhizosheath and the rhizosphere microbial community in growing media. Ammonia oxidation is a major process in nitrogen cycling, as it is the first limiting reaction for overall rate of nitrate production. Ammonium is one of the principal plant absorbable N forms. Thus, nitritation and its control by plants may play a central role in the outcome of competition for nitrate and ammonium between plants and microbial communities^28^.

The aim of this study was to determine the effect of two recovered nutrients used as nitrogen sources, namely struvite and organic fertilizer, on tomato plant performance, nitrogen dynamics in the growing medium, microbial community associated with the rhizosheath and rhizosphere over time, and abundance and functionality of nitrogen turnover-associated microbes, such as AOB and NOB.

We hypothesized that (a) the availability of the nutrients from the struvite will rely mainly on the chemical release rate (i.e. dissolution changing because of pH, time, water content), while organic fertilizer will require microbes to become available; that (b) the overall microbial community will be affected by the presence of a plant in comparison to bulk soil, and by the fertilizer treatment, due to root processes related to nutrient uptake that may influence microbial community structure and activity; and that (c) the fertilizer effects are significant for nitrogen-related bacteria, as we would expect larger impact from organic fertilizer (boosting heterotrophs and nitrifiers) vs. struvite (boosting only nitrifiers).

## Results

### Organic fertilizer resulted in higher biomass and leaf area

Organic fertilizer led to a 41% higher tomato biomass compared with struvite at time point 2, 20 days after sowing (DAS; average plant biomass of 0.21 g for organic and 0.16 g for struvite). Biomass of plants under both N treatments was greater than no-N treated plants (average of 0.02 g). The differences between fertilizers were smaller at time point 3 (34 DAS), but the organic fertilizer still led to significantly higher biomass (*P* < 0.05) than struvite (average of 3.1 g with organic and 2.2 g with struvite). Fertilizer (*P* < 0.05) and time point (*P* < 0.05) had significant effects not only on fresh and dry weight but also on leaf area. The largest mean leaf area (990.3 cm²) was found when organic fertilizer was provided (Table S1).

### N dynamics in the growing medium-rhizosphere-plant

Recovered nutrients distinctly impacted the N-dynamics, as ammonium and nitrate concentration of the growing medium varied over time. Ammonium and nitrate concentrations in the growing medium were significantly influenced by the presence of plant (*P* < 0.01), fertilizer (*P* < 0.05) and time point (*P* < 0.05). In the treatments with plants, ammonium concentration increased continuously in the struvite treatment (Table 1). Conversely, when the organic fertilizer was provided, ammonium concentration was reduced while nitrate concentration increased at time point 2. Similarly, the ammonium concentration was further decreased in the organic treatment at time point 3 (second harvest), but nitrate concentration was also reduced, indicating potential nitrate-N plant uptake (Table 1). In the growing medium without plants, ammonium turnover was not significantly different in comparison with the same treatment in the tomato plants.

**Table 1:** Influence of fertilizer type (no fertilizer -NOF, organic fertilizer – ORG and struvite-STR) on the nutrient dynamics (pH, electrical conductivity (EC), ammonium and nitrate) in non-sterile organic growing medium with plants (Tomato) and without plants (No plant) as a function of time.

Time point	Plant	Fertilizer	NH_4_^+^-N (mg L^−1^)	NO_3_^–^N (mg L^−1^)	P (mg L^−1^)	pH (H_2_O)	Conductivity (µS/cm)
1: Starting exp.	-*	NoFert	1.7 ± 1.4	0.0 ± 1.03	18.7 ± 4.9	5.5 ± 0.03	93.3 ± 6.7
		Organic	18.4 ± 1.4	0.0 ± 1.03	27.3 ± 4.9	5.7 ± 0.03	127.3 ± 6.7
		Struvite	42.1 ± 1.4	0.0 ± 1.03	203. ± 4.9	5.6 ± 0.03	83.0 ± 6.7
2: First Harvest (20 DAS)	No plant	NoFert	3.6 ± 1.4	0.0 ± 1.03	12.9 ± 4.9	6.2 ± 0.03	98.7 ± 6.7
		Organic	20.1 ± 1.4	2.5 ± 1.03	21.6 ± 4.9	6.2 ± 0.03	130.9 ± 6.7
		Struvite	49.4 ± 1.4	0.0 ± 1.03	150.1 ± 4.9	6.1 ± 0.03	181.5 ± 6.7
	Tomato	NoFert	5.0 ± 1.4	0.0 ± 1.03	10.3 ± 4.9	5.9 ± 0.03	123.5 ± 6.7
		Organic	3.6 ± 1.4	23.3 ± 1.03	18.2 ± 4.9	5.7 ± 0.03	199 ± 6.7
		Struvite	42.1 ± 1.4	0.0 ± 1.03	126.8 ± 4.9	6.0 ± 0.03	194.4 ± 6.7
3: Second harvest (34 DAS)	No plant	NoFert	0.4 ± 1.4	0.0 ± 1.05	11.5 ± 4.9	6.0 ± 0.03	141.3 ± 6.7
		Organic	16.3 ± 1.4	13.8 ± 1.05	19.7 ± 4.9	5.6 ± 0.03	251.2 ± 6.7
		Struvite	54.7 ± 1.4	13.1 ± 1.05	206.1 ± 4.9	5.6 ± 0.03	284.4 ± 6.7
	Tomato	NoFert	1.9 ± 1.4	0.0 ± 1.05	9.5 ± 5.0	5.7 ± 0.03	189.4 ± 6.7
		Organic	0.5 ± 1.4	3.1 ± 1.05	18.3 ± 5.0	5.5 ± 0.03	327 ± 6.7
		Struvite	74.3 ± 1.4	0.0 ± 1.05	290.7 ± 5.0	5.6 ± 0.03	332.4 ± 6.7

n = 5. Tpt 1 = 0 days after sowing, Tpt 2 = 20 days after sowing and Tpt 3 = 34 days after sowing. Average ± error of the mean. * : at the start of the experiment the growing medium was analyzed.

Shoot N concentrations analysed in plant tissue (mg N 100 mg^−1^ plant) was in the normal range in the first and second harvesting, as defined by Marschner^29^. No significant differences between fertilizers were detected at any time point (4.5% N from struvite and 4% N from organic fertilizer in the second harvest). P concentration was significantly higher with the struvite (0.95% versus the 0.66% measured with the organic fertilizer at second harvest).

N uptake of plants treated with organic fertilizer was higher than struvite treated plants, as indicated by the mg of N in the shoot tissue of tomato plants. Biomass response showed a similar trend. Plants treated with organic fertilizer yielded a higher biomass in the first and second harvest compared with the struvite fertilizer treatment. However, the absolute differences decreased over time. The percentage of N remaining in the substrate at time point 3 was 74% of the applied N when struvite was applied. In contrast, the N measured in the growing medium in the organic fertilizer treatment was 3.6% of the applied N. The total N recovered, i.e. the total nitrogen content measured in the two tomato plants per rhizotron, was nearly 20% with the struvite, and 25% with the organic fertilizer, respectively (Table S2).

### pH dynamics in the bulk, rhizosphere, and rhizosheath

**Figure 2:**
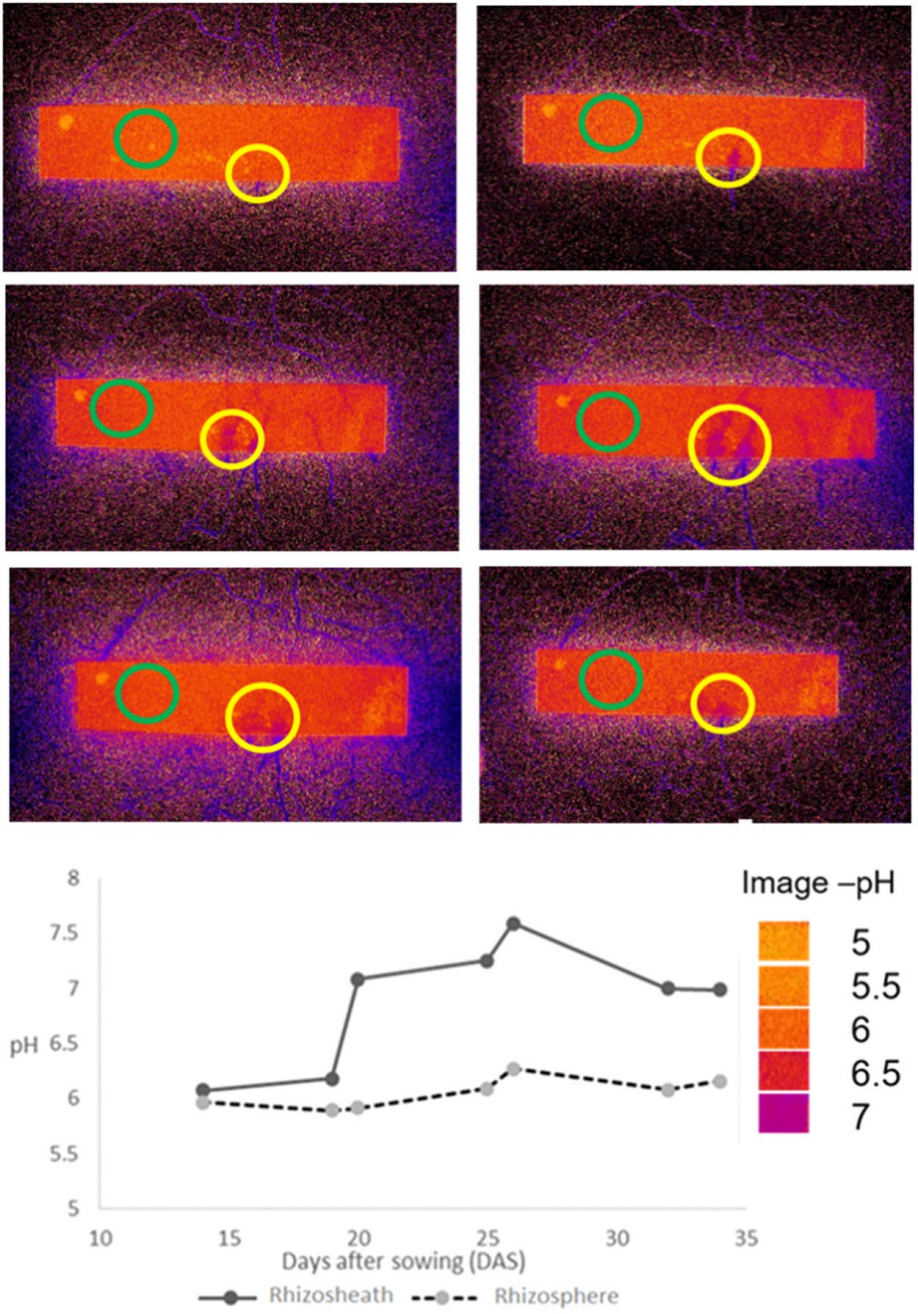
Sequence of pH changes in the rhizosphere of the tomato plants along six measurements with the upper pH optodes under the organic fertilizer treatment (from left to right and top to bottom). Green circles show where the pH values were determined for the rhizosphere, and yellow circles show where the pH values were determined for the rhizosheath. Shades of red denote pH values lower than 6.5, shades of blue-purple denote pH increase from 7 up to 7.5. The graph represents the corresponding pH values after the calibration made with 8 known pH values that allowed to calculate the equation to extrapolate the values obtained with the Image J program to the real pH values. Solid line represents the values for the rhizosheath (yellow circles) and dashed line represents the values for the rhizosphere (green circles). The change in color from red to purple indicates an increase in pH. The increased pH value is measured after the calibration in the rhizosheath reaching a maximum pH of 7.5, 26 days after sowing the tomato plant, and decreasing again to a value of pH 7, 35 days after sowing, when the plants were harvested.

The pH (H_2_O) of the growing medium measured in a 1:5 v/v water extract was significantly influenced by plant, fertilizer and time point (*P* < 0.05, Table 1). The pH was monitored in the rhizosphere and rhizosheath via the installed planar optodes (Fig. 2). Distinctive pH patterns were only found with organic fertilizer (n = 2 out of 10). We observed increased pH in the rhizosheath when the root was crossing the optode (Fig. 2). The rhizosphere pH changed from 6.2 to 7.6. This pH decreased over time to 6.9 at the second harvest (34 days after sowing). The pH of the rhizosphere increased to a value of 6.2, confirming the pH value measured in the growing medium with a pH-meter (H_2_O).

### Relative microbial abundance as affected by fertilizer, plant, and age (time)

**Figure 3:**
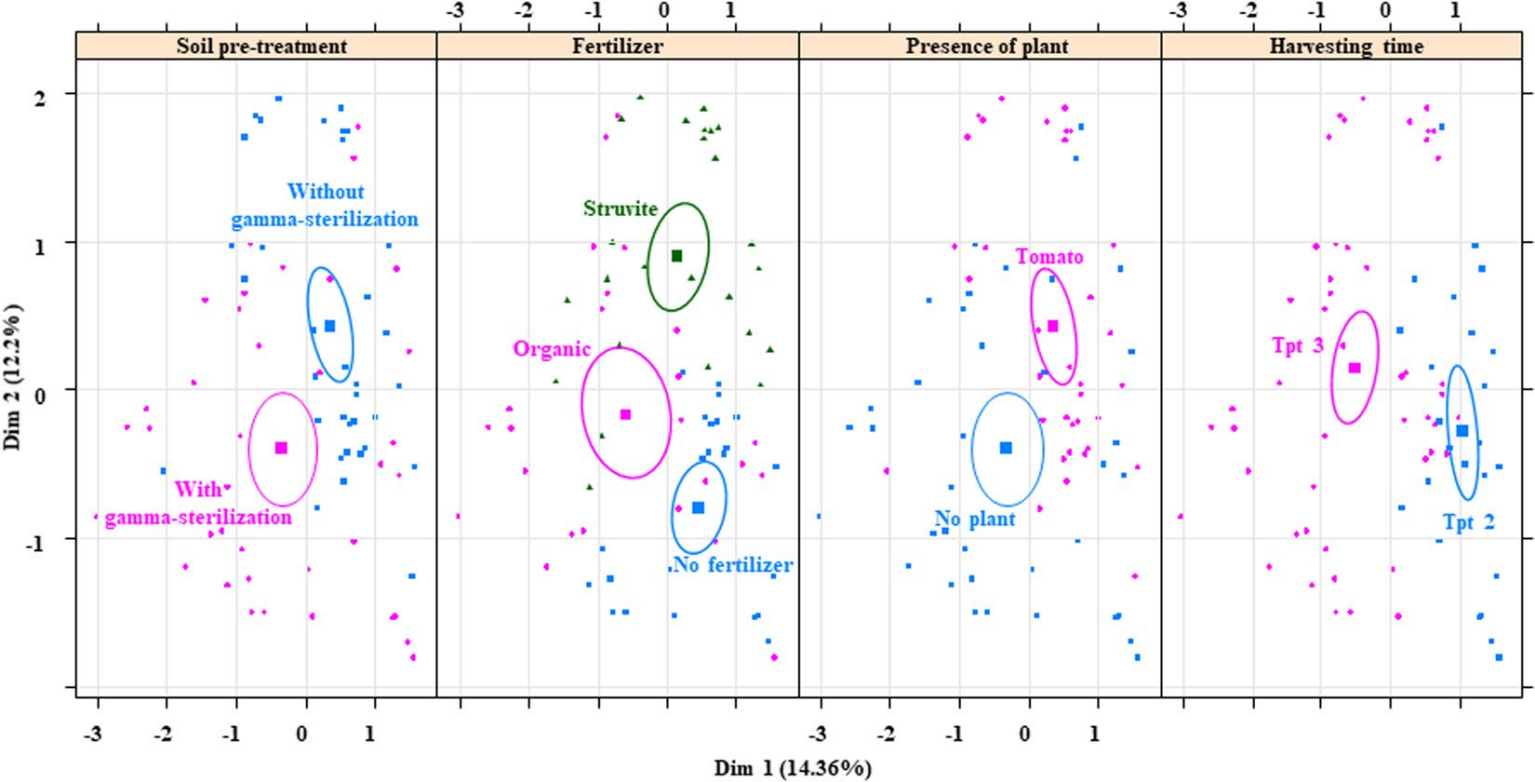
Microbial community shifts of pre-treated bulk growing media harbouring tomato plants, supplemented with fertilizer and followed over time. Multiple Factor Analysis (MFA) revealed variations in the relative bacterial abundances and ellipses show confidence Intervals (CI) of 95% for each sample type. Confidence ellipses (CI = 95%) revealed that the impact of plant occurrence, time and sterilization on the microbial community was greater than that of fertilizer, showing a dissimilarity of the relative abundances because of plant presence. The first dimension of the MFA described the soil without fertilizer and harbouring tomatoes, while the second dimension was constructed by the variables associated with the soil supplemented with struvite and harbouring tomato plants. Each dot in the plot indicates one sample.

Multiple Factor Analysis (MFA) was employed to detect how the plant presence and fertilizers contributed to the differences between relative abundances of bacterial genera in the growing media across time. Relative abundances of each sample were weighted, and these weights are identical for the relative abundances of the same group (time/plant/fertilizer). Tomato and the no fertilizer treatment in the rhizosphere at time point 2 accounted for 14.36% of the variance in relative abundances among all the samples (Dimension 1, *P* < 0.0001). Tomato and the struvite in the rhizosphere contributed to 12.2% of the variance (Dimension 2, *P* < 0.0001, Fig. 3). Confidence ellipses (CI = 95%) revealed that the impact of plant occurrence, time and sterilization on the microbial community was greater than that of fertilizer, showing a dissimilarity of the relative abundances because of plant presence (Fig. 3).

**Figure 4:**
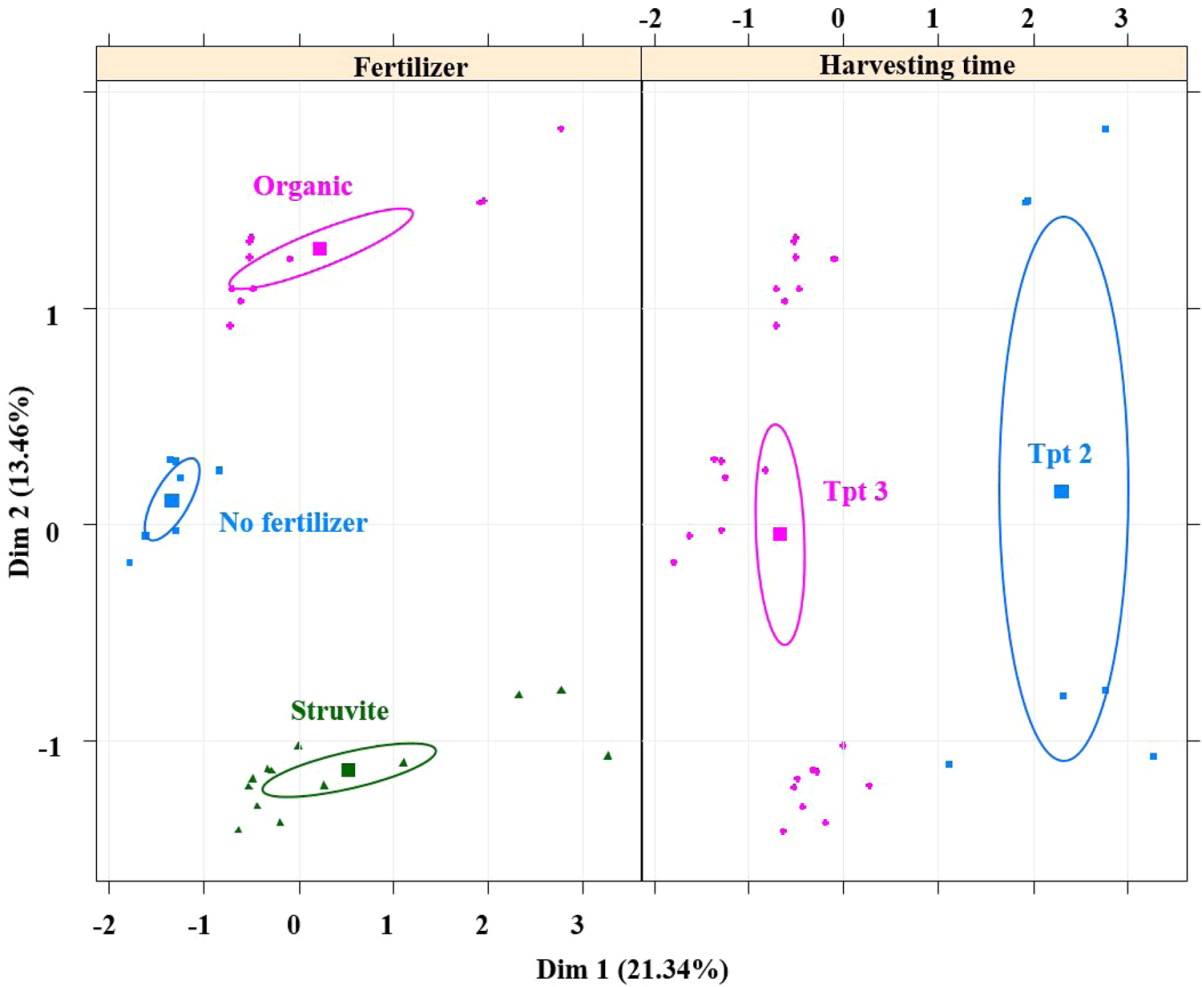
Microbial community shifts of rhizosheath (less than 1 mm from root) of tomato plants, supplemented with fertilizer and followed over time. Multiple Factor Analysis (MFA) revealed variations in the relative bacterial abundances and ellipses show confidence Intervals (CI) of 95% for each sample type. MFA was independently performed for the relative abundances detected in the rhizosheath showing that the fertilizer (fer) and time point (tpt) significantly influenced the relative abundances of bacterial genera in the tomato rhizosheath. The first dimension of the MFA described the growing medium fertilized with struvite at time point 2 (Tpt 2), while the second dimension was constructed by the variables associated with the growing medium supplemented with the organic fertilizer. Plots indicate that fertilizer influences the relative abundances of bacterial genera in the tomato rhizosheath. Each dot in the plot indicates one sample.

**Figure 5:**
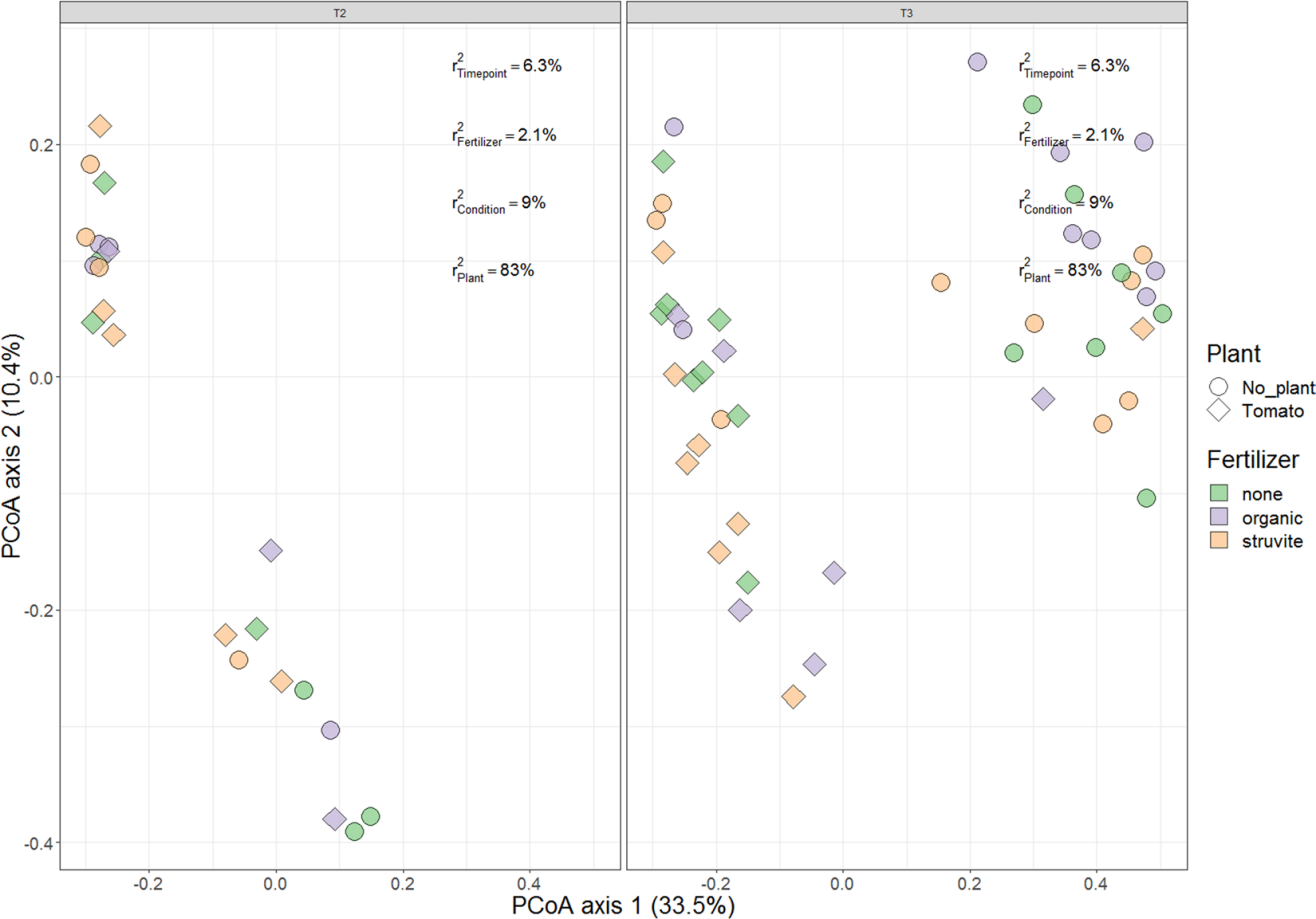
Beta diversity of rhizosphere bacterial communities in growing media with or without tomato plants. Circles indicate communities from rhizosphere harbouring tomato plants, and diamonds show bulk growing medium without plants. Purple indicates communities in growing medium that were supplemented with organic fertilizer, while orange indicates addition of struvite and green is no fertilizer application. Samples of gamma-irradiated soil were followed over time to observe the bacterial community development. The growing medium harbouring plants was not sterile, while the samples collected at time point 2 and 3 were from the sterile growing medium without plants. Thus “condition” indicated whether the growing medium was sterile or remained with its native bacterial community. PERMANOVA results indicate that plant is the factor explaining the highest percentage of the variance among communities in the rhizosphere.

**Figure 6:**
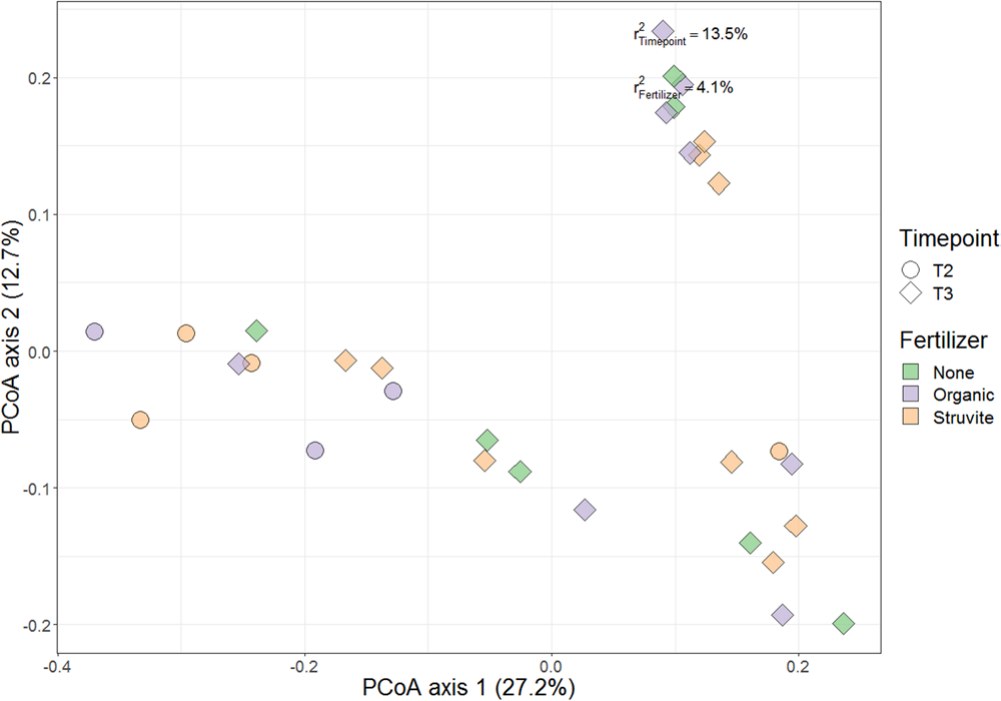
Beta diversity of rhizosheath bacterial communities in growing media with or without tomato plants. Circles indicate communities from the rhizosheath at time point 2, while diamonds indicate bacterial communities at time point 3. Purple indicate rhizosheath communities supplemented with organic fertilizer, while orange shows the communities harbouring tomato plants, supplemented with organic fertilizer, and green is for those without fertilizer. Samples of gamma-irradiated growing media were followed over time to observe the bacterial community development. The figure shows that the community structure in the rhizosheath was not significantly different as a result of fertilizer applied.

MFA was independently performed for the relative abundances detected in the rhizosheath (Fig. 4), showing that fertilizer and time point significantly influenced the relative abundances of bacterial genera in the tomato rhizosheath (Fig. 4). Analysis of multivariate homogeneity of group dispersions (PERMANOVA) was performed to indicate the significance of each covariate (time, fertilizer and gamma-sterilization) on the microbial community of the bulk. Communities in growing medium without fertilizer remained similar throughout the experiment (Fig. 5), while those in growing medium supplemented with organic fertilizer were dissimilar at time point 3 (P < 0.05). PERMANOVA was also performed to indicate the significance of each covariate (time and fertilizer) on the microbial community of the rhizosheath. The fact that the composition was not changed (Fig. 6) suggests that the diversity and evenness remained constant, but the relative abundance of each genus was different between communities, following fertilizer supplementation (Fig. S3, Tables S3 and S4).

### Microbial community structure is influenced by fertilizer type

Community metrics were not significantly different as a result of location, i.e. rhizosheath versus rhizosphere, which means that the number of species (richness), the relative abundances of each of these species (evenness) and the pool of species (diversity) was the same in the rhizosheath and rhizosphere. However, fertilizer impacted alpha diversity and evenness not richness (Table 2, Fig. S2). Microbial community evenness tended to be higher when no fertilizer was applied.

**Table 2:** Effect of location (rhizosheath and rhizosphere), fertilizer (no fertilizer, organic and struvite) on microbial species richness (total species), diversity (Shannon, Fisher’s alpha, Simpson and Inverse Simpson indices), and evenness (Pielou’s index) for the third time point ( = second harvest) (n = 81).

	Location (mean ± SEM)		Fertilizer (mean ± SEM)			Effect *(P*-value)		
	Rhizosheath	Rhizosphere	NOF	ORG	STR	Location	Fertilizer	Location* Fertilizer
Total species	274 ± 10	292 ± 9	301 ± 12	286 ± 11	262 ± 11	NS	NS	NS
Pielou	0.552 ± 0.002	0.549 ± 0.002	0.554 ± 0.002^b^	0.551 ± 0.002^a^	0.546 ± 0.002^c^	NS	*	NS
Shannon	3.08 ± 0.02	3.1 ± 0.02	3.2 ± 0.02^a^	3.1 ± 0.02^b^	3.00 ± 0.02^c^	NS	**	NS
Simpson	0.7456 ± 0.0002	0.7454 ± 0.0002	0.74^63^ ± 0.0003^a^	0.7457 ± 0.0003^b^	0.7445 ± 0.0003^c^	NS	**	NS
Fischer Alpha	59.0 ± 1.6	61.7 ± 1.6	65.2 ± 2.1^a^	61.3 ± 1.9^b^	54.5 ± 1.9^c^	NS	**	NS
Inverse Simpson	3.931 ± 0.003	3.929 ± 0.003	3.942 ± 0.004^a^	3.933 ± 0.004^b^	3.914 ± 0.004^c^	NS	**	NS

Tomato (n = 45); NOF = no fertilizer (n = 25); ORG = organic fertilizer (n = 27) and STR = struvite (n = 29), rhizosphere (n = 40; rhizosheath (n = 41). Different letters in each row indicate significant differences between treatments based on Tukey HSD post-hoc multiple comparison. ns, *,** indicates non-significant (ns) or significant at the 0.05 (*) or 0.001 (**) probability level, respectively.

### Functional bacterial groups in the rhizosphere are directly impacted by recovered nutrients

Quantification of the *amoA* gene was used as a benchmark for tracking the abundance of total ammonium-oxidizing bacteria, using qPCR. Total copy number per g of growing medium from tomato plants was significantly influenced by fertilizer treatment (*P* < 0.05, Table 3) and was not significantly influenced by location at the second harvest (Table 3). The lowest *amoA* copy number was recorded when no fertilizer was applied; no differences were found between struvite and the organic fertilizer (Table 3). The location had significant effect on total bacteria, total Archaea, *Nitrobacter*, and *Nitrospira* (*P* < 0.05, Table 3). Total bacteria were higher in the rhizosheath compared to the rhizosphere, except for *Nitrospira*, where the opposite was observed.

**Table 3:** Influence of fertilizer type (no fertilizer - NOF, organic fertilizer – ORG and struvite - STR) and the location (rhizosphere versus rhizosheath) on the total and the relative amount of copy numbers of different microbial groups per g of growing medium in non-sterile organic growing media with tomato. N = 8. NS = no significant effect. Numbers are µ ± σ; LOD = limit of detection was 10^3^ for amoA bacteria, 16S Bacteria, 16S Archaea, 16S Nitrobacter and 16S Nitrospira and 10^2^ for the archaeal amoA gene copy number. LOD = limit of detection. Different letters in each row indicate significant differences between treatments based on Tukey HSD post-hoc multiple comparison. ns, *,** indicates non-significant (ns) or significant at the 0.05 (*) or 0.001 (**) probability level, respectively.

Microbial groups	Fertilizer*			Location		Effect (*P*-value)		
	NF	ORG	STR	Rhizosphere	Rhizosheath	Fertilizer	Location	Location *Fertilizer
16S Bacteria	1.0 × 10^8^ ± 4.8 × 10^7^	2.0 × 10^8^ ± 4.8 × 10^7^	2.5 × 10^8^ ± 4.6 × 10^7^	1.3 × 10^8^ ± 4.6 × 10^7^	2.4 × 10^8^ ± 3.9 × 10^7^	NS	*	NS
16S Archaea	1.6 × 10^7^ ± 7.2 × 10^6^	2.9 × 10^7^ ± 7.2 × 10^6^	3.5 × 10^7^ ± 6.9 × 10^6^	1.7 × 10^7^ ± 5.8 × 10^6^	3.5 × 10^7^ ± 5.8 × 10^6^	NS	**	NS
*amoA* Bacteria (AOB)	<LOD^a^	5.1 × 10^6^ ± 1.0 × 10^6b^	1.4 × 10^6^ ± 1.7 × 10^6b^	2.3 × 10^6^ ± 1.6 × 10^6^	4.2 × 10^6^ ± 1.3 × 10^6^	*	NS	NS
Nitrobacter (NOB)	1.7 × 10^6^ ± 8.7 × 10^5^	3.2 × 10^6^ ± 8.7 × 10^5^	3.3 × 10^6^ ± 8.4 × 10^5^	1.4 × 10^6^ ± 7.0 × 10^5^	4.1 × 10^6^ ± 7.0 × 10^5^	NS	*	NS
Nitrospira (NOB)	3.7 × 10^4^ ± 3.1 × 10^4^	1.2 × 10^5^ ± 2.8 × 10^4^	1.3 × 10^5^ ± 3.1 × 10^4^	5.6 × 10^4^ ± 2.2 × 10^4^	1.4 × 10^4^ ± 2.5 × 10^4^	NS	*	NS
*amoA Bacteria*/16SBacteria	<LOD^a^	2.6 ± 0.3%^b^	0.7 ± 0.8%^a^	1.3 ± 0.7%	2.0 ± 0.5%	*	NS	NS

The relative AOB abundance (ratio copy number of bacterial *amoA*: total bacteria) was significantly impacted by fertilizer type (*P* < 0.05), but not by location or interaction between location and fertilizer. AOB was 100 times higher in the organic fertilizer in comparison with the no fertilizer treatment and the struvite (Table 3). The archaeal *amoA* gene copy number was below the detection limit of 2.5 × 10^3^ gene copy numbers per µL (Table 3). The relative NOB abundance (ratio of the (*Nitrobacter* + *Nitrospira*): total bacteria) was not significantly influenced by fertilizer and location (data not shown). *Nitrobacter* was higher in absolute and relative numbers in comparison to *Nitrospira* within the NOB community in the organic growing medium. No significant shifts in the relative *Nitrobacter/Nitrospira* ratio associated with the location, fertilizer treatment or the interaction of both were detected (Table 3).

### Potential ammonia oxidation rate is decreased in the rhizosheath compared to the rhizosphere

The *ex situ* potential ammonia oxidation rate determined after the 2^nd^ harvest, showed that the activity per location was significantly higher in the rhizosphere (*P* < 0.001). Significant effects of the fertilizer treatments were found and were lower (*P = *0.05) when no fertilizer treatment was applied (31.9 ± 3 µg N g growing medium^−1^ day^−1^). However, no significant differences were found between the organic fertilizer (36.6 ± 3 µg N g growing medium^−1^ day^−1^) and struvite (37.3 ± 2 µg N g growing medium^−1^ day^−1^). Longitudinal analysis showed that the potential activity of the ammonia-oxidizing bacteria in the rhizosheath was 38% lower than in the rhizosphere (60.0 ± 4 µg N g growing medium^−1^ day^−1^).

## Discussion

We hypothesized that the availability of struvite will rely mainly on the chemical release rate, while organic fertilizer will require microbes to convert the organically bound nitrogen to ammonium and to make it available for the plant. Our results show that both struvite and organic are suitable fertilizers as they can deliver plant available nutrients. However, the use of struvite resulted in decreased leaf area, fresh weight, and dry weight compared to the organic fertilizer. We found that the ammonium from the struvite seemed to be released faster than that from the organic fertilizer. However, the ammonium concentration decreased in the growing medium with organic fertilizer, indicating that most of the ammonium was either used by the plant or was mineralized. Higher biomass production is normally obtained by a combined presence of both ammonium and nitrate^29^. Indeed, the organic fertilizer treatment showed a combined presence of ammonium and nitrate and the highest growth rates and plant yields. In contrast, no nitrate was detected with the struvite treatment, indicating that ammonium was not transformed to nitrate or was immediately taken up by the plant. The availability of nitrogen from struvite is a combination of a chemically and biologically driven process^30^. Struvite has slow-release properties^31^ determined by the dissolution rate and solubility^32^. The small increase in the concentration of nitrate observed when struvite was applied to the medium without plants can be explained by the presence of bacteria, altering the dissolution rate of ammonium from struvite^33^. This rate may ultimately impact the physicochemical characteristics of the growing medium and/or microbial community, and therefore plant performance^34,35^.

The increase of rhizosphere pH observed with the optodes indicated plant nitrate uptake only with the organic fertilizer. It is possible that pH changes were not visualized in other treatments and replicates because pH was not continuously monitored but only every second day. However, these preliminary results agree with the nutrient turnover analyses, indicating that the tomato plant was more effective in taking up nitrogen from organic fertilizer than from struvite. Struvite supply resulted in a steadily net increase of the ammonium concentration over time, most likely due to higher dissolution rates compared to ammonia oxidation rates^34,36^. This might result in high or even toxic ammonium concentrations and subsequently decreased plant performance^34^.

We found evidence that bacterial abundance between samples of the rhizosphere of tomato and bulk without plant were significantly different, as expected. When plants start rooting, they immediately encounter the microbial community associated with the growing medium, resulting in the establishment of a rhizosphere community closely interacting with the plants and distinct from the bulk growing medium^37,38^. In our study, the community composition in the rhizosphere and bulk growing medium without a plant was distinctly different indicating that plants can influence the microbial community composition even at a distance of more than 10 mm in organic growing media.

In addition, we found significant differences between microbial community structure and activity in the rhizosphere and rhizosheath. Indeed, the total number of species (richness), the relative abundances of each of these species (evenness) and the pool of species (diversity) at each time point/on each fertilizer/with or without plant was the same in the rhizosphere and rhizosheath. However, differences in community composition and species composition between the rhizosphere and rhizosheath indicated that not all the same species were present in all environments (Tables S3 and S4). Thus, the number of species may be equal but turnover between species may be high and the way they are distributed within time points, within plants, and within fertilizers may be different.

The colonization of the rhizosphere is the result of a complex exchange of signals between the microorganisms and the plant, which can be beneficial to plants^39^. Indeed, microorganisms can affect plant-nutrient acquisition processes by influencing nutrient availability in the rhizosphere and/or functionality of the biochemical mechanisms underlying the nutritional process^39^. We found significantly increased relative abundance of *Asticcacaulis*, *Opitutus*, *Sphingomonas* and Uncultured *Fibrobacter* in the rhizosphere, which could be linked to the organic fertilizer treatment. Ambrosini, *et al*.^40^ showed plant growth promoting characteristics of putatively diazotrophic bacteria from the rhizosphere and *Asticcacaulis* was associated with the rhizosphere. *Sphingobacteriales* are copiotrophic bacteria with high cellulose degradation activity towards a wide range of carbon sources and are present in soils or growing media with high organic carbon content^41^. The carbon sources are likely to come from the growing medium as it contained high organic matter (34.9% of the dry matter is cellulose)^42^. Similar to our findings, tomato plants inoculated with endophytic *Sphingomonas* sp. LK11 (Sphingobacteriales) showed significantly increased plant growth, and shoot dry weight compared to the control^43^ (Table S1). Moreover, as Sphingobacteriales require oxygenated environments for carbon degradation, the decreased relative abundance of Sphingobacteriales in the struvite treatment might indicate a lack of oxygen, explaining decreased nitrification rates when struvite was supplied. Relative abundance of *Acidothermus*, a genus belonging to Acidobacteria, was higher when struvite was provided. This group of bacteria is mostly considered oligotrophic (K-strategist) and decline with increasing N concentrations^44^. We found the opposite probably because Acidobacteria cannot use the unavailable crystalized NH_4_^+^ present as struvite. *Azospirillum*, *Bdellovibrio*, *Rhizomicrobium* and Uncultured Planctomycetaceae had significant higher relative abundance when no fertilizer was applied. This can be explained since *Azospirillum* induces changes in plant root architecture, promoting the development of lateral and adventious roots and root hairs^39^. Moreover, *Bdellovibrio* spp. is a bacterial genus known for unique predatory behaviour, as they attack other Gram negatives, penetrate their periplasm, multiply in their cytoplasm, and finally burst their cell envelopes to start anew, resulting in a release of plant-available nutrients^45^.

Differences in the microbial community structure of the rhizosphere were mainly a result of time (Fig. 5), which has been described for maize^46^. Plant root exudates are differentially produced at distinct development stages, orchestrating rhizosphere microbiome assemblage^47^ and exudation rate increases during the juvenile growing stage as the plant grows^22,46,48,49^. Consequently, spatiotemporal differences are important factors influencing rhizosphere microbial community composition. Bacterial communities in the rhizosheath were significantly different as a result of fertilizer supplementation, which was not observed in the rhizosphere. MFA showed that the microbial community in the rhizosheath becomes stable over time, as indicated by the decreased variations in the bacterial relative abundances over time. Consequently, differences in rhizosheath microbial community composition compared to the rhizosphere are mainly influenced by plant presence and time, i.e. plant age or developmental stage.

Absence of fertilizer treatment resulted in higher species richness (total species), diversity (Shannon, Fisher’s alpha, Simpson and Inverse Simpson indices), and evenness (Fig. S2). Many dominant bacterial groups might be dormant under particular rhizosphere conditions, but their presence would still be detected by DNA-based analyses. Under P and N deficient conditions plant shoot and root biomass are decreased compared with optimal conditions and consequently, root exudation and influence on rhizosphere microbial community composition may be decreased. Plants produce a root exudate zone adjacent to and just behind root tip meristems^50^, thus altering numbers and diversity of microbes on root surfaces and in the rhizosheath and rhizosphere^51^. Exudates produced in nutrient-limited media (i.e. without fertilizer/struvite) are increased, probably leading to increased microbial activity around roots and increased nutrient ‘microbial mining’^52^. Through the secretion of root exudates, plants may be considered to be gardening microorganisms^51^.

Plant roots activate mineralization of organic nitrogen^53^ leading to increased ammonium fluxes in the rhizosphere^8^. The uptake of ammonium^29,34^ by the roots suggests a direct competition for ammonium with the AOB in the rhizosphere. The nitrogen originating from the organic fertilizer is supplemented as organic nitrogen, while the nitrogen coming from the struvite is supplemented as ammonium. These results indicate organic nitrogen mineralization activity as a result of the organic fertilizer supplementation and higher nitrification activity in comparison to that in struvite. Nitrate was produced only when organic fertilizer was supplied, but its absence in the struvite treatment may indicate inhibition of the nitrification activity. Nitrate accumulates in the growing medium either under high nitrification rates and/or low nitrate reduction rates, i.e. low microbial immobilization, denitrification or dissimilative nitrate reduction to ammonium (DNRA)^44^. The heterotrophic microbial community regulates whether nitrogen is lost or retained in the growing medium^54–56^, thus organic nitrogen mineralization relies directly on the microbial nitrogen conversion and results in a release of ammonia, which is subsequently oxidized by NOB into nitrate. The growing medium supplemented with the organic fertilizer showed decreased pH values compared to the growing medium blended with struvite, indicating increased acidotolerant nitrification activity through heterotrophic nitrification^57^, but also through autotrophs^58^. In addition, the organic nitrogen mineralisation results not only in a release of ammonia, but also of CO_2_^59^ an indispensable carbon source for the autotrophs. AOB abundance was not affected by location but by fertilizer used. Indeed, we found a higher biodiversity in the organic fertilizer compared to the struvite, while no differences were found in the log number of *amoA* AOB copies per g of dry growing medium between the organic fertilizer and struvite. Lower AOB numbers were observed when no fertilizer was applied, suggesting that AOB benefit from the increased N supply by the fertilizers and not from the increased N supply from roots, efficiently competing for the ammonia with other microorganisms and/or with the tomato plant^8^. Competition relies on the ammonium availability and diffusion of the different N fertilizers in the growing medium and we found significant differences in nitrogen dynamics between treatments.

Ammonium availability impacts both the nitrification rates and the nitrifier population size^60^. Hence, affinity for ammonium might be a key characteristic in the rhizosheath. Microorganisms with high affinity for ammonium have low growth rates and are classified as K-strategists. On the contrary, microorganisms with a low affinity have in general a high growth rate and are classified as r-strategists^61^. The results from Table 3 might indicate that the rhizosheath is mainly colonized by K-strategists able to compete with the plants. The rhizosphere, on the contrary, might be more colonized by r-strategists due to the decreased competition with the plant and showing a higher growth rate and maximum ammonia oxidation activity. Higher microbial activity in the rhizosphere, including organic nitrogen mineralization, may stimulate ammonia oxidizing bacteria (AOB) and archaea (AOA). Ammonia uptake by plants may favour AOA, considered to prefer lower ammonia concentrations, while high ammonia concentrations may favour AOB^8^. In general, microorganisms are superior to plants with respect to the competition for nitrogen^62^. Our results suggest that AOB are better competitors for ammonia/ammonium than plants under fertilized conditions (100 mg N L^−1^ growing medium), but plants are the better competitors under N deficient conditions. Within the NOB community in the organic growing medium, *Nitrobacter* seems to be a key player in absolute and relative numbers compared to *Nitrospira*. *Nitrobacter* is a superior competitor when resources are abundant, while *Nitrospira* thrives under conditions of resource scarcity^63^. We found no shifts in the relative *Nitrobacter/Nitrospira* ratio associated with location and fertilizer treatment, indicating interactions between ammonia oxidizers and nitrite oxidizers^64^.

Functionality tests revealed that the potential ammonia oxidation activity was significantly higher (*P* < 0.05) in the rhizosphere in comparison with the rhizosheath, indicating inhibition of the ammonia oxidation in the rhizosheath or even stimulation of ammonia oxidation in the rhizosphere. Non-invasive pH measurements with the planar optodes showed an increase of the rhizosphere pH and the presence of nitrate with the organic fertilizer. Indeed, uptake of nitrate results in excess uptake of anions over cations, net uptake of protons and thus an increase in the rhizosheath/rhizosphere pH^29^. We found increased pH values in the rhizosheath/rhizosphere indicating that nitrification rates were not inhibited by acidification^65,66^. Plant roots can release compounds to suppress nitrification (biological nitrification inhibition)^67^. Inhibition of nitrification is likely to be part of an adaptation mechanism to conserve and use N efficiently in natural systems that are N limiting^68,69^. However, this was not shown in soilless culture systems with organic growing medium and tomato plants. In addition, nitrification inhibition is stimulated in the presence of ammonium^67^ and we found higher ammonium concentrations in the struvite treatment.

## Conclusion

Our results shed light on how roots and microorganisms orchestrate, coexist and benefit from each other especially at juvenile plant growing stage, although they depend on the same nutrients and strongly compete for them, particularly in the rhizosphere. Tomato plants seem to influence or even modulate the nitrification activity in the rhizosphere and the highest relative AOB abundance was found with the organic fertilizer. This was confirmed by the increase over time in the community evenness, indicating that plants rather than fertilizers are shaping microbial community composition. Furthermore, the effect of the plant on the microbial community is observed in the activity of the N-related bacteria. As the ammonia oxidation rate was reduced in the rhizosheath, the plant may be impacting the activity of the ammonia oxidizing bacteria to compete for the N sources.

Based on our results, ammonium seems to be the key N form for successful nitrogen competition in soilless culture systems. The paramount reason for efficacious nitrogen acquisition from N hotspots in soilless culture systems is spatiotemporal and plant-mediated differences in nitrogen availability and in overall and specific microbial community distributions. There is no doubt that generating a detailed understanding of rhizosheath-rhizosphere related microbial community, their assembly over time and activity will be essential to manipulate root-soil interactions and to ensure sustainable fertilizer use-efficiency and soilless crop production in the future.

## Methods

### Experimental setting, growing medium and recovered nutrients used

The struvite used in this study was recovered from wastewater (provided by The Laboratory of Chemical and Environmental Engineering, Lequia, University of Girona, Spain). The commercially available organic fertilizer (Frayssinet, France), consists of vegetable and animal-based material, sugar beet vinasse, cake, and fruit pulp and composted manure. The animal protein is processed in accordance with the regulations of EC 1069/2009 (hydrolyzed powder of feathers, bones and meat, horn meal and dried blood powder). The chemical composition of the recovered nutrients contained in the fertilizers used is detailed in Table S5. The organic growing medium (GB, Grow Bag, Greenyard, Belgium) consisted of a mixture of white peat (H2-H4 on the von Post scale^70^ [40% v/v], Irish peat [40% v/v] and coconut fiber [20% v/v]). The average fresh bulk density (n = 4) of the growing medium was 225.04 kg/m³, determined according to EN12580. The growing medium had a gravimetric water content of 0.50 ± 0.02 kg kg^−1^. Fertilizers were mixed with the organic growing medium at a dose of 100 mg N L^−1^ growing medium (equivalent to 300 kg N ha^−1^ in arable soil) suitable for sowing tomatoes^71^.

Tomato plants (*Solanum lycopersicum* L. × *Solanum habrochaites* Maxifort, Monsanto Vegetable Seeds, Bergschenhoek, The Netherlands) were grown in rhizotrons with dimensions of 60 cm × 30 cm × 2 cm filled with the organic growing medium. Growing plants in rhizotrons allow combined analyses of root architecture and visualization of rhizosphere pH changes. All rhizotrons were placed in climate chambers at the Institute of Plant Sciences (IBG-2; Forschungszentrum Jülich GmbH, Jülich, Germany) under the following controlled conditions: day length of 16 h, day/night temperatures of ~24/18 °C, and illumination was <400 μmol m^−2^ s^−1^ between 06:00 and 22:00 hours local time.

Each rhizotron (Fig. 1) was filled with 1.1 kg of the growing medium, equaling approx. 5 L. The total number of rhizotrons was 60, with 10 replicates of each treatment (struvite, organic and no N fertilization; each of them with and without tomato plants). The growing medium on the control treatment was sterilized using gamma–irradiation (BGS, Wiehl, Germany) at minimal dose of 50 kGy to eliminate the native microbial community. The use of gamma irradiation as a method for growing medium sterilization for laboratory experiments has been recommended over other sterilization techniques^72^.

The rhizotrons had one removable side of transparent polycarbonate plate so that planar pH-optodes (Presense GmbH, Regensburg, Germany) could be installed^73^ (Supplementary Information: Material and Methods). To ensure that the nutrient supply was adequate for plant growth for the duration of the experiment, all essential nutrients other than N were provided via application of 1 L of modified 1/3 Hoagland nutrient solution to a final concentration of (in g L^−1^) 0.01 P, 0.5 S, 1.2 Ca, 0.016 Mg, and 1.2 K.

Time point 1 was considered as the time when the rhizotrons were filled and the seedlings were transplanted into the rhizotron. When the roots reached the centre of the first optode about 20 days after sowing, 50% of all the rhizotrons were opened and the first harvest was done to collect the microbial and growing media samples (i.e. time point 2). Two weeks after the first harvest, the remaining 50% of the rhizotrons were harvested (i.e. time point 3) to collect the microbial and growing media samples. The experiment ended at this point because the plant roots reached the bottom of the rhizotrons. Shoot fresh weight and leaf area were determined at time point 2 and time point 3 (Supplementary Information: Material and Methods).

### Physicochemical analysis of the growing medium and plant tissues

Chemical/physical characteristics (pH, electrical conductivity, and total nutrient content) of the growing medium were determined at all time points and correlations were evaluated (SAS, version 9.4, SAS Institute, Cary, USA). Nutrients were extracted (1:5 v/v) in ammonium acetate and measured with ICP-OES (Table S8). The electrical conductivity (EC) and pH, ammonium (NH_4_^+^) and nitrate (NO_3_^−^) were measured in a 1:5 v/v water extract according to EN 13038, EN 13037 and EN 13652, respectively. Nitrate was measured with a Foss Fiastar 5000 continuous flow analyzer. Ammonium was measured by steam distillation.

Nutrient contents of plant samples were determined by element analysis via inductively coupled plasma optical emission spectrometry (ICP-OES; VarioELcube, Elementar, Langenselbold, Germany). The pH of the growing medium was determined using standard electrodes (Hanna Instruments pH 209 pH meter, Vöhringen, Germany), using 1:5 distilled water extract at 20 °C.

### Detection of pH dynamics in the rhizosphere with planar optodes

In this experiment, the planar optodes (Fig. 1) were used for non-invasive *in situ* measurement of pH dynamics in the rhizosphere to link pH changes with microbial community structure and nutrient turnover. The used planar optodes had a measuring threshold between pH(H_2_O) = 5.5 and 8.30. The setup and methodology for the use of the optodes is explained in the Supplementary Information: Material and Methods. The calibration curve to extrapolate measured values from the optodes to pH values is shown in Fig. S1. The calibration was done by using six different conventional pH buffer solutions, ranging from 4 to 9 (Riedel-de Haën; Sigma-Aldrich Laborchemikalien GmbH, Seelze, Germany) which makes it possible to convert the value of the optode measurement into real pH values. Pictures of the two optodes in each rhizotron (n = 5) for all treatments were taken every second day to visualize pH changes.

### Sampling of growing media for nutrient concentration and microbial community composition analyses

Substrate samples were collected in the zone of the upper pH sensitive optodes. We took samples in the bulk zone (i.e. rhizotrons containing only growing medium without plants) and at two distances to the root: a) “rhizosphere” (region at approximately 1 cm distant from the root) and b) “rhizosheath” samples taken directly at the root (less than 1 mm distance to a root) (Fig. 1).

Samples from the rhizosheath and rhizosphere zone were collected using tweezers and scalpels sterilized with 70% ethanol. To collect samples from the rhizosheath, roots passing the optode were cut at the upper and lower zone of the optode with a sterilized scalpel to avoid contamination and to collect only the roots in the optode zone. The fresh weight of the growing medium adhering to the roots was quantified. All samples for chemical and microbial analyses were taken in triplicate per zone sampled per rhizotron (n = 3). The fresh weight of each sample was determined, and samples were immediately stored at −80 °C for microbial community analysis.

In total, 203 samples were collected for determination of the microbial community composition, of which 194 samples were selected for sequencing. Total DNA was extracted from the growing medium samples using the Power Soil^®^ DNA Isolation Kit (MoBio Laboratories Inc., Carlsbad, CA, USA), following the manufacturer’s instructions. We used 500 mg from the rhizosphere and 100 mg from the rhizosheath. Concentration and quality of DNA were measured based on the absorbance at 260 and 280 nm in a Nanodrop ND 1000 spectrophotometer (NanoDrop Technologies, Wilmington, DE, USA).

### Quantification of total bacteria, Archaea, NOB (*Nitrobacter* sp. and *Nitrospira* sp.), AOB and AOA

Quantitative PCR assays of 49 samples from both rhizosheath and rhizosphere were completed using an ABI StepOnePlus real-time PCR system. Reactions were performed in a total volume of 20 μl, with 10 μl of 2x iTaq universal SYBR Green Supermix (Bio-Rad Laboratories, Hercules, CA, USA), 1 μl of DNA template (50 ng μL^−1^), 1 μl of each primer (Table S6) and nuclease-free water volume adapted according to the primer concentration used. Table S7 gives an overview of the target microbial community (total bacteria (519F and 907R), total Archaea (Arch349F and Arch806R), AOA (crenamo23f and crenamo616r) AOB (amoA1F and amoA2R), NOB (NSR1113F, NSR1264R, Nitro-1198F and Nitro-1423R)) and the template used. Amplifications were run as follows: initial denaturation for 2 min at 95 °C, followed by 40 cycles of 15 s denaturation at 95 °C, 30 s annealing at a specific annealing temperature and 30 s extension at 60 °C. At the end of the qPCR run, a melting curve analysis was performed to confirm product specificity (60–95 °C, ΔT per 15 s = 0.3 °C). Quantification was performed using a standard curve based on known concentrations of DNA standard dilutions from 10^7^ copies μl^−1^ to 10^2^ copies μL^−1^. Each sample was quantified in triplicate.

### Microbial community structure assessment

High-throughput amplicon sequencing of the V3–V4 hypervariable region^74^ was performed with the Illumina MiSeq platform according to the manufacturer’s guidelines at LGC Genomics GmbH (Berlin, Germany). Contigs were created by merging paired-end reads based on the Phred quality score (of both reads) heuristic as described by Kozich, *et al*.^75^ in Mothur^76^ (v.1.33.3). Contigs were aligned to the SILVA database and filtered from those with (i) ambiguous bases, (ii) more than 8 homopolymers, and (iii) those not corresponding to the V3–V4 region, which resulted in a removal of 75% of the sequences. The sequencing errors were removed using IPED, an algorithm dedicated to de-noise MiSeq amplicon sequencing data^77^. Chimera removal was performed using the CATCh tool^78^ set in *de novo* mode, which resulted in the removal of an additional 16% of the sequences. Detailed information about classification, clustering and quality of the sequencing can be found in Supplementary Information: Material and Methods.

Richness and alpha diversity using the Fisher’s diversity, Shannon, Simpson, and inverse Simpson indices were calculated within each sample (Supplementary Information: Material and Methods).

Beta diversity estimates based on Chao and Bray-Curtis indices were used to examine dissimilarity and determine the impact of experimental factors on microbial community structure. Principal Coordinate Analysis (PCoA) was employed to visualize the differences among samples, using the vegan package in R^79^. Stratified permutational multivariate analysis of variance (PERMANOVA) with 999 permutations was conducted to indicate the significance of time, fertilizer and plant presence on the microbial community. ANOVA was applied to reveal whether the distribution of the genera was different when the plant was present^79^. Because of the over-dispersion in the OTU data, a zero-inflated count model was used to assess the effect of fertilizer and plant and the interactions between plant*fertilizer on each individual bacterial genus, in both rhizosphere and rhizosheath sampling zones (Supplementary Information: Material and Methods). The final model was selected based on the Akaike Information Criterion (AIC). Differences among library size sample were accounted for with the offset option in proc GLIMMIX in SAS^80^. *P* values for each comparison were converted to q-values that were then used to identify differences in relative abundances of bacterial genera while controlling false discovery rate (FDR) at the 5% level^81^.

### *Ex-situ* tests for the potential ammonia oxidation activity tests

In addition to the determination of the microbial community composition, samples for *ex situ* activity tests were collected at time point 3 (i.e. 2^nd^ harvest, 34 days after sowing). High-throughput batch activity tests were performed^82^ to determine the potential ammonia oxidation rate in the rhizosheath and rhizosphere from the organic growing medium without fertilizer and with organic fertilizer or struvite, respectively. For this analyses, four samples of each treatment (no fertilizer, organic or struvite), with a total of 60 samples, were collected in the rhizosphere/rhizosheath observed with the first installed planar optode. As an internal control, samples were collected from the gamma-sterilized growing medium. The rhizosheath and rhizosphere samples (weighing each 0.132 ± 0.078 g) were stored for 48 h at 21 °C before the batch activity tests were started. Samples were subsequently mixed with a P-buffer to a final ratio of 30 mg of growing medium per mL of buffer and vortexed for 1 min at maximum speed. The buffer solution (pH 6.5) further contained final concentrations of 0.774 g P L^−1^ (KH_2_PO_4_/K_2_HPO_4_), 0.1 g NaHCO_3_ L^−1^ and 25 mg N L^−1^ as (NH_4_)_2_SO_4_. Then, six replicates of 260 µL each were transferred to 96-well plates and incubated in a MB100-4A Thermo shaker (Hangzhou Allsheng Instruments, China) at room temperature, at 600 rpm and sealed with parafilm (Benis NA, Neenah, WI, USA) to minimize evaporative losses. Ammonium concentrations were determined using a Tecan infinite M200 PROplate reader (Männedorf, Switzerland), following the Berthelot reaction^83^.

### Multivariate statistical analysis for evaluating relationships between the microbial community and growing medium features

Differences in physicochemical characteristics among growing medium supplemented with the different fertilizers were compared using a mixed model in SAS (5 version 9.4, SAS Institute, Cary, USA). Pearson correlations (Tables S8, S9, S10) were used to determine the interactions between the physicochemical characteristics and significance was assumed at *P* < 0.05. Eleven variables were included in the analysis: pH(H_2_O), conductivity, nitrate-N, ammonium-N, phosphorus, potassium, calcium, magnesium, sulfate, sodium, chloride. Multiple Factor Analysis (MFA) was used to detect how the relative abundances of bacterial genera differed in growing medium harbouring either tomato plants or no plants. The function MFA from the FactoMineR package^84^ was performed in R. Parametric bootstrapping was applied to construct confidence ellipses around the barycenter of the samples included on each covariate (time/fertilizer/plant presence), and thus visualize whether the bacterial abundances were significantly different among any of these categorical descriptors. If the ellipses were not overlapping, the bacterial abundances were significantly different; incomplete overlap indicated that bacterial abundances were significantly different in the samples outside the ellipse^85^. Statistical differences in ammonia oxidation rate were analyzed using a longitudinal mixed model in SAS. A random slope model was used with time point, fertilizer and location (bulk, rhizosphere or rhizosheath) as fixed factors and all interactions were considered. Six technical replicates (meaning repetitions within one replicate) were nested within each biological replicate (n = 4). Unstructured covariance structure was used, assuming that the variance differed between rhizosheath and rhizosphere.

### Accession codes

All MiSeQ data have been deposited in GenBank and are available under the accession no. PRJNA521111.

## Supplementary information

Supplementary Information
