# Supplementary material for: Tomato plants rather than fertilizers drive microbial community structure in horticultural growing media
Source: Sci Rep. 2019 Jul 2;9:9561. doi: 10.1038/s41598-019-45290-0 (PMC6606572; doi:10.1038/s41598-019-45290-0)

Supplementary Information

Tomato plants rather than fertilizers drive microbial community structure in horticultural growing media

Oliver Grunert[1,2+], Ana A. Robles-Aguilar[3+], Emma Hernandez-Sanabria[1], Silvia D. Schrey[3], Dirk Reheul[4], Marie-Christine Van Labeke[4], Siegfried E. Vlaeminck[1,5], Tom G. L. Vandekerckhove[1], Mohamed Mysara[6,7], Pieter Monsieurs[6], Vicky M. Temperton[8], Nico Boon[1]*, and Nicolai D. Jablonowski[3]*

[1]Center for Microbial Ecology and Technology (CMET), Ghent University, Coupure Links 653, 9000 Gent, Belgium
[2]Greenyard, Skaldenstraat 7a, 9042 Desteldonk, Belgium
[3]Forschungszentrum Jülich GmbH, Institute of Bio- and Geosciences, IBG-2: Plant Sciences, 52428 Jülich, Germany
[4]Department of Plant and Crops, Ghent University, Coupure Links 653, 9000 Gent, Belgium
[5]Research Group of Sustainable Energy, Air and Water Technology, Department of Bioscience Engineering, University of Antwerp, Groenenborgerlaan 171, 2020 Antwerpen, Belgium
[6]Unit of Microbiology, Belgian Nuclear Research Center (SCK•CEN), Mol, Belgium
[7]Department of Bioscience Engineering, Vrije Universiteit Brussel, Brussels, Belgium
[8]Institute of Ecology, Leuphana University Lüneburg, Universitätsallee 1, D-21335 Lüneburg, Germany

+these authors contributed equally to this work.

*shared principal investigator and corresponding authorship:

Prof. Dr. Nico Boon, CMET: Center for Microbial Ecology and Technology - FBE - Ghent University, Coupure Links 653, 9000 Gent, Belgium, Phone: +32 (0)9 264 59 76, e-mail: Nico.Boon@UGent.be

Dr. Nicolai D. Jablonowski, Institute of Bio- and Geosciences, IBG-2: Plant Sciences, Forschungszentrum Jülich GmbH, 52425 Jülich, Germany, Phone +49-2461-61-8682 Fax -2492, e-mail: n.d.jablonowski@fz-juelich.de

Supplementary Information

Supplementary Information: Materials and Methods section

Experimental Setting

Tomatoes were germinated on filter paper before transplanting them as 2 days old seedlings (2 seedlings of tomatoes per rhizotron). The seedlings were planted at a depth of 2 cm and in contact with the Plexiglas. Rhizotrons were maintained at an angle of 45° during the growing period to ensure the maximum number of visible roots growing along the glass.

To maintain a growing medium water content of ~30% (volumetric water content) 60 mL deionized water was applied 3 times per week.

Assessment of leaf area, fresh and dry weight of tomato plants

Shoot fresh weight and leaf area were determined at time point 2 and time point 3. Fresh weight was measured directly after harvesting (Mettler Toledo XS205, Gießen, Germany). Subsequently, leaf area was determined using a leaf area meter (Li- 3100, Li-cor, Nebraska, USA). Plant samples were dried at 65 °C in a forced-draft oven until dry weights were stable.

Setup and methodology for measurements with the optodes

Planar optodes offer a unique opportunity to monitor *in situ* the concentration changes of the analyte of interest in the vicinity of roots based on photoluminescence[59]. The planar optode set-up consisted of a foil containing the sensor fixed to the transparent inner side of the rhizotron surface, which comes into contact with roots and the associated rhizosphere[60]. The sensor foils with embedded fluorescent molecules emitting a characteristic pattern of fluorescence after excitation depending on the analyte concentration[58,63,64].

One optode was placed on the glass with special glue (GE Bayer Silicone, Leverkusen, Germany) at 27 cm from the top, and the second optode was placed at a depth of 16.5 cm measured from the

first optode or 43 cm from the top. The optodes had a pH-sensitive side directed to the growing medium, whereas the glue side was directed to the glass. To place the optodes, the glass was removed carefully from the rhizotron, keeping it at horizontal position. After placing the optodes, it was screwed back onto the rhizotron.

A camera sensitive to the emission range of the optode detects this fluorescence signal, which serves as an information carrier. Further, using light as an information carrier allows for separation of the sensor (i.e. the planar optode) and detector (the camera).

Planar optodes can also be used for guided sampling and pH measurement in the rhizosphere[62] to determine optimal sampling times based on the observation of strong pH changes; and locations based on the specific area where pH changes were observed. In this approach, optodes did not indicate time and place of sampling, as the harvest was done when the root crossed the first optode independently of the observations.

Sampling of growing medium

To sample the rhizosphere and rhizosheath, the Plexiglas plates with the attached optodes were carefully removed from the rhizotron without destroying the roots. A paper tissue with holes at the location of the optodes and the size of the Plexiglas plate was placed on the rhizotron leaving open spaces of identical size to the optodes. The sampling zone for the microbial community analysis thus equaled the surface of the optodes.

Microbial community structure assessment

Sequences were classified using the RDP trainset[70] version 9, removing those with Eukaryota, Mitochondria or Chloroplast classifications. The sequences were clustered into operational taxonomic units (OTUs) at 97% identity level with UPARSE[71] on default settings (v7.0.1001_i86linux32) via the *sortbysize*, *cluster_otus*, and *usearch_global* commands. Quality of the sequencing and post-processing pipeline was verified by incorporating mock samples (n = 12 species) in triplicate into the same sequencing run. A total of 778,411 reads were obtained. After examining read-counts, if any Operational Taxonomic Unit (OTU) was not classified up to the genus level, the consensus sequence was blasted using the SILVA database v1.2.11 to obtain the

taxonomic classification[72]. Singletons that remained unclassified were culled.

Pielou's index was used as an indicator of evenness in the community. Differences in alpha diversity and evenness measures among treatments were compared using a mixed model in SAS (version 9.4, SAS Institute, Cary, USA), with fertilizer (no fertilizer, organic fertilizer or struvite), plant (no plant or tomato), location (rhizosheath versus rhizosphere) as a fixed effect for the third time point. Hence, the differences in the diversity measures at the end of the experiment could be attributed to plant, fertilizer, and location or to the interaction of the three factors.

ANOVA was applied to reveal whether the distribution of the genera was different whenever the plant was present[73]. Because of the over-dispersion in the OTU data, a zero-inflated count model was used to assess the effect of fertilizer and plant and the interactions between plant*fertilizer on each individual bacterial genus, in both rhizosphere and rhizosheath sampling zones. Zero-inflated models explain the excess of zeros by modeling the data as a mixture of a Poisson distribution or a negative binomial distribution. When a zero count is observed, there is the zero-inflation probability, because the observation came from the always-zero distribution. When the underlying count distribution is a Poisson distribution, the model is called a zero-inflated Poisson distribution and if the count distribution is a negative binomial distribution, the mixture is called a zero-inflated negative binomial distribution. The final model was selected based on the Akaike Information Criterion (AIC). Differences among library size sample were accounted for with the offset option in proc GLIMMIX in SAS[74]. P values for each comparison were converted to q-values that were then used to identify differences in relative abundances of bacterial genera while controlling false discovery rate (FDR) at the 5% level[75].

Table S1: Influence of fertilizer type (no fertilizer - NOF, organic fertilizer – ORG and ammonium struvite - STR) on the performance of tomato plants in a non-sterile organic growing medium as a function of time. n = 5. Tpt 1 = time point 1 (i.e. experiment start, no plant, growing medium only); Tpt2 = time point 2 (i.e. harvest 1, 20 days after sowing) and Tpt 3 = time point 3 (i.e. harvest 2, 34 days after sowing). NA = not applicable, (P <0.05) SEM = standard error of the mean. ns, *,** indicates non-significant (ns) or significant at the 0.05 (*) or 0.001 (**) probability level, respectively.

Variable	Tpt	Fertilizer			SEM	P value		
		NOF	ORG	STR		Fertilizer	Time point	Fertilizer* Time point
Leaf area(cm²)	0 DAS	NA	NA	NA	46.0	**	**	**
	20 DAS	6.64	182.3	102.1				
	34 DAS	95.3	990.3	734.9				
Fresh weight (g)	0 DAS	NA	NA	NA	1.83	**	**	**
	20 DAS	0.14	3.92	2.16				
	34 DAS	2.46	35.18	25.22				
Dry weight (g)	0 DAS	NA	NA	NA	0.191	**	**	**
	20 DAS	0.01	0.208	0.16				
	34 DAS	0.24	3.08	2.24				

Table S2: Recovery of N by tomatoes fertilized with different nitrogen sources (organic fertilizer and struvite) in organic growing medium at two harvests (20 and 34 days after sowing - DAS). N_{tot} recovered shows the total nitrogen content measured in the two tomato plants per rhizotron. N_{min} growing medium shows the concentration of N (ammonium and nitrate) in the growing medium. NOF = no fertilizer; ORG = organic fertilizer and STR = struvite. LOD = limit of detection. Each number is the mean value of n = 5 ± standard deviation.

Treatment	Timepoint	N_{tot} recovered (%)	N_{min} growing medium (%)
NOF	20 DAS	< LOD	< LOD
ORG		2.7±0.5	26.8±3.4
STR		1.5±0.4	42.1±9.7
NOF	34 DAS	1.2±0.3	1.9±0.0
ORG		24.6±12.6	3.6±0.0
STR		19.8±7.8	74.0±0.0

Table S3: Relative abundances of bacteria genera in the rhizosheath of tomato plants detected at the final harvest (time point 3, n = 12). Analysis was performed separately for rhizosheath and rhizosphere to consider time effect and to uncover whether the final bacterial relative abundance was impacted only by fertilizer and not by time. The bacterial relative abundance values indicated in the table are for those genera that were not significantly different at the end of the experiment, but only between fertilizers. Thus, differences in relative abundance was attributed to fertilizer effect.

**Taxonomy**	**Fertilizer (mean ± SEM)**									***P* value**
	None			Organic			Struvite			
3x genus Incertae Sedis	0.940	±	0.103	0.439	±	0.101	0.586	±	0.170	0.008
Aciditerrimonas	0.002	±	0.002	0.003	±	0.002	0.015	±	0.006	0.02
Acidobacteria Gp14	0.010	±	0.010	0.010	±	0.003	0.024	±	0.006	0.03
Acidocella	0.040	±	0.022	0.019	±	0.003	0.042	±	0.010	0.04
Acidothermus	0.013	±	0.001	0.020	±	0.009	0.051	±	0.013	0.001
Adhaeribacter	0.016	±	0.007	0.003	±	0.003	0.004	±	0.002	0.005
Afipia	0.114	±	0.011	0.238	±	0.073	0.230	±	0.040	0.02
Anaeromyxobacter	0.078	±	0.002	0.043	±	0.008	0.046	±	0.005	0.001
Azospirillium	0.507	±	0.058	0.292	±	0.063	0.279	±	0.041	0.02
Bdellovibrio	0.318	±	0.057	0.192	±	0.037	0.228	±	0.036	0.05
Brevundimonas	0.027	±	0.028	0.004	±	0.003	0.014	±	0.007	0.04
Cryptosporangium	0.025	±	0.005	0.041	±	0.003	0.051	±	0.013	0.04
Dokdonella	0.002	±	0.001	0.002	±	0.002	0.009	±	0.005	0.04
Flavobacterium	0.0002	±	0.0002	0.003	±	0.003	0.001	±	0.001	0.02
Gallionella	0.044	±	0.015	0.009	±	0.007	0.015	±	0.006	0.002
Gemmata	0.022	±	0.009	0.016	±	0.008	0.032	±	0.015	0.05
Kofleria	0.109	±	0.022	0.039	±	0.019	0.032	±	0.014	0.004
Novosphingobium	0.123	±	0.019	0.081	±	0.023	0.079	±	0.017	0.001
Pseudonocardia	0.001	±	0.001	0.001	±	0.001	0.007	±	0.004	0.01
Rhizomicrobium	6.485	±	0.576	4.164	±	0.254	5.083	±	0.851	0.03
Rhodocyclaceae	0.002	±	0.001	0.0004	±	0.0003	0.004	±	0.003	0.003

Stigmatella	0.056	±	0.006	0.016	±	0.005	0.030	±	0.008	0.008
Telmatospirillum	0.046	±	0.013	0.036	±	0.007	0.034	±	0.014	0.04
Unclassifed Chlamydiales	0.033	±	0.016	0.005	±	0.002	0.011	±	0.007	0.03
Unclassified Gammaproteobacteria	0.420	±	0.067	0.271	±	0.034	0.266	±	0.025	0.04
Unclassified Sandaracinus	0.001	±	0.0004	0.005	±	0.004	0.003	±	0.001	0.01
Uncultured Planctomycetaceae	0.723	±	0.094	0.514	±	0.071	0.671	±	0.201	<0.0001
Vampirovibrio	0.011	±	0.001	0.006	±	0.003	0.004	±	0.002	0.002
Zavarzinella	0.013	±	0.006	0.006	±	0.006	0.007	±	0.003	0.02

Table S4: Relative abundances of bacteria genera in the rhizosphere of tomato plants detected at the final harvest (time point 3, n = 12). Analysis was performed separately for rhizosheath and rhizosphere to consider time effect and to uncover whether the final bacterial relative abundance was impacted only by fertilizer and not by time. The bacterial relative abundance values indicated in the table are for those genera that were not significantly different at the end of the experiment, but only between fertilizers. Thus, differences in relative abundance was attributed to fertilizer effect.

**Taxonomy**	**Fertilizer (mean ± SEM)**									***P* value**
	None			Organic			Struvite			
Achromobacter	0.0007	±	0.0005	0.0004	±	0.0003	0.0033	±	0.0040	0.006
Archangium	0.0461	±	0.0136	0.0775	±	0.0169	0.0887	±	0.0201	0.04
Asticcacaulis	0.14	±	0.0467	0.2661	±	0.0211	0.2744	±	0.1066	0.04
Beijerinckia	0.0108	±	0.0040	0.0385	±	0.0294	0.0243	±	0.0149	0.01
Byssovorax	0.0039	±	0.0039	0.0025	±	0.0029	0.0006	±	0.0003	0.03
Cytophaga	0.0021	±	0.0014	0.0067	±	0.0034	0.0078	±	0.0051	0.01
Iamiaceae	0.0003	±	0.0002	0.0036	±	0.0014	0.0016	±	0.0011	0.0003
Opitutus	0.0428	±	0.0103	0.1842	±	0.0616	0.0497	±	0.0154	0.001
Phaselicystis	0.0034	±	0.0030	0.0059	±	0.0019	0.0035	±	0.0028	0.02
Polyangium	0.0022	±	0.0014	0.0110	±	0.0050	0.0061	±	0.0044	0.03
Pseudomonas	0.0239	±	0.0123	0.0019	±	0.0013	0.0037	±	0.0020	0.003
Rhodopirellula	0.0004	±	0.0003	0.0038	±	0.0028	0.0011	±	0.0005	0.0006
Rickettsia	0.0007	±	0.0005	0.0003	±	0.0002	0.0019	±	0.0020	0.0004
Sediminibacterium	0.0019	±	0.0013	0.0047	±	0.0031	0.0054	±	0.0057	0.003
Sphingomonas	0.0985	±	0.0345	0.3274	±	0.2456	0.1264	±	0.0582	0.003
Telmatospirillum	0.0100	±	0.0046	0.0463	±	0.0076	0.0161	±	0.0045	0.003
Terrimonas	0.0014	±	0.0009	0.0039	±	0.0024	0.0074	±	0.0056	0.008
Unclassified *Sandaracinus*	0.0025	±	0.0028	0.0018	±	0.0010	0.0004	±	0.0002	0.01
Uncultured *Fibrobacter*	0.0001	±	0.0001	0.0013	±	0.0012	0.0005	±	0.0002	0.0008

Table S5: Chemical composition of the recovered nutrients. ND = not determined. stdev = standard deviation

Parameters	Organic fertilizer (mean ± stdev)	Struvite ($NH_4MgPO_4\ 6H_2O$) (mean ± stdev)
Total N (%)	7.78 ± 0.19	ND
Organic-N (%)	6.89 ± 0.17	ND
NH_4-N (%)	0.36 ± 0.02	6.6±0.2
NO_3-N (%)	0.017 ± 0.001	ND
Urea-N (%)	0.51 ± 0.03	ND
P in mineral acid (%)	2.19 ± 0.06	13.2± 0.4
K in water (%)	4.93 ± 0.12	ND
Ca total (%)	5.70 ± 0.14	ND
Mg total (%)	0.57 ± 0.03	10.6± 0.2
S total (%)	2.14 ± 0.05	ND
Na total (%)	0.48 ± 0.02	ND
Organic matter (%)	54.4 ± 1.4	ND

Table S6: Overview of the primer concentration, volume and annealing temperature used for the specific bacterial groups.

	Primer concentration	*Volume*	*Annealing temperature*
16S Bacteria	*500 nM*	*0.5µL*	*57°C*
16S Nitrobacter	*300 nM*	*0.3µL*	*64°C*
16S Nitrospira	*600 nM*	*0.6 µL*	*54°C*
amoA Bacteria	*500 nM*	*0.5 µL*	*59°C*
16S Archaea	*800 nM*	*0.8 µL*	*60°C*
AmoA archaea	*500 nM*	*0.5 µL*	*59°C*

Table S7: Overview of the target bacteria and the template used.

Target	Template (stD curve)		reference
**16S bacteria**	PCR product *E. coli* ATCC 10536	519F	
		907R	
**16S Archaea**	PCR product *Archaeoglobus fulgidus* DSM4304	Arch349F	
		Arch806R	
**16S Nitrospira**	PCR product from 16S Nitrospira (environmental sludge) clone	NSR 1113F	(Dionisi et al. 2002)
	Company DANIS (manure treatment)	NSR 1264R	(Graham et al. 2007)
**16S Nitrobacter**	PCR product from 16S Nitrobacter (nitrifying bacterial consortium) clone	Nitro-1198F	
	ABIL (AVECOM)	Nitro-1423R	
**amoA Bacteria**	PCR product from amoA (*N. europaea*) clone	amoA 1F	(Rotthauwe et al. 1997)
		amoA 2R	
**amoA Archaea**	PCR product from fosmid 54D9	crenamo23f	
		crenamo616r	(Treusch et al. 2005)

Table S8: Correlations (r) among chemical characteristics and plant performance (leaf area, fresh weight, dry weight and dry matter content) in organic growing medium for cultivating tomato plants, with struvite fertilizer (n = 5). In parenthesis, the time point when the correlation was observed. ***P < 0.0001, **P < 0.05.

	**Conductivity**	**P**	**K**	**SO_4^{2-}**	**Na**	**Cl**	**Leaf area**	**Fresh weight**	**Dry weight**	**Dry matter content (%)**
**pH(H2O)**	-0.969** (2)									
**Conductivity**			0.908** (3)		-0.879** (3)	0.962**(3)				0.926** (2)
**NH_4^+-N**		0.988** (2)								
**P**			-0.929** (3)			-0.901** (3)	0.992*** (2)<br>0.978**(3)	0.918**(2)	0.901**(2)	0.946** (3)
**K**				0.951** (3)		0.967** (3)	-0.912** (3)			-0.923** (3)
**Leaf area**								0.937** (2)	0.927** (2)	
**Fresh weight**									0.970** (2)	

Table S9: Correlations (r) among chemical characteristics and plant performance (leaf area, fresh weight, dry weight and dry matter content in organic growing medium for cultivating tomato plants, with organic fertilizer (n = 5). In parenthesis, the time point when the correlation was observed. ***P < 0.0001, **P < 0.05.

	**Mg**	**SO_4^{2-}**	**Cl**	**Fresh weight**	**Dry weight**
**Conductivity**		0.964*** (3)	0.948** (2)<br>0.979** (3)		
**Ca**	0.881** (3)				
**Cl**				-0.889** (2)	
**Fresh weight**					0.948**(2)

Table S10: Correlations (r) among chemical characteristics and plant performance (leaf area, fresh weight, dry weight and dry matter content) in organic growing medium for cultivating tomato plants, without fertilizer (n = 5). In parenthesis, the time point when the correlation was observed. ***P < 0.0001, **P < 0.05.

	**Conductivity**	**P**	**K**	**SO_4^{2-}**	**Cl**	**Leaf area**	**Fresh weight**	**Dry weight**
**pH(H2O)**	-0.982** (2)			-0.993*** (2)	-0.980** (3)			
**Conductivity**			0.977**(3)	0.903** (3)	0.999*** 2)			
**NH_4^+-N**		0.881** (3)						
**P**			-0.913** (2)					
**Ca**						-0.916** (3)		
**SO_4^{2-}**					0.975** (2)			
**Leaf area**							0.974** (2)<br>0.915** (3)	0.915**(2)
**Fresh weight**								0.995***(3)

Figure S1: Calibration curve to extrapolate measured values from the optodes to pH values. Rm is the measured R-value, i.e., the ratio of red to green in the emitted fluorescence response (Gansert and Blossfeld 2008, Progress in Botany, 333-358, DOI: 10.1007/978-3-540-72954-9_14).

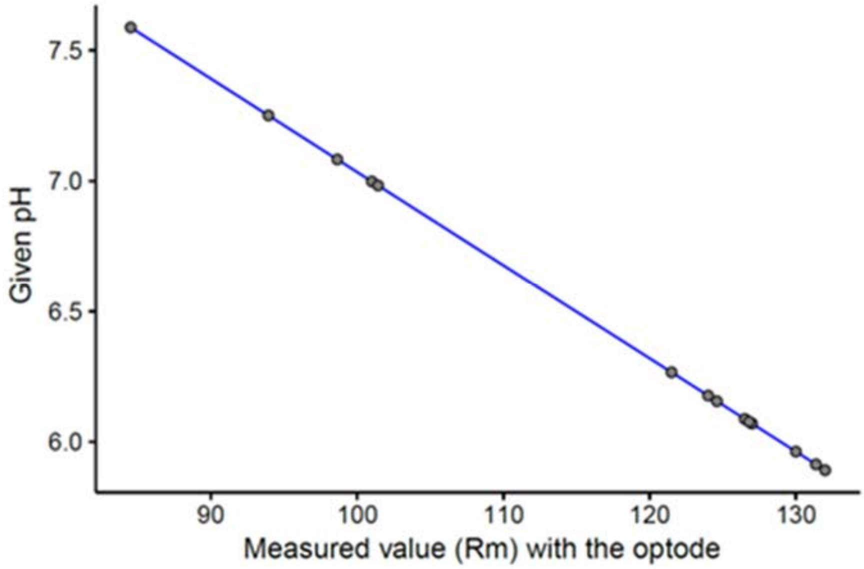

Figure S2: Condition of the growing medium and plant presence were determinants for impacting community characteristics: alpha diversity in the rhizosheath (A) and rhizosphere (B), observed diversity (number of species) in rhizosheath (C) and rhizosphere (D) and evenness in rhizosheath (E) and rhizosphere (F).

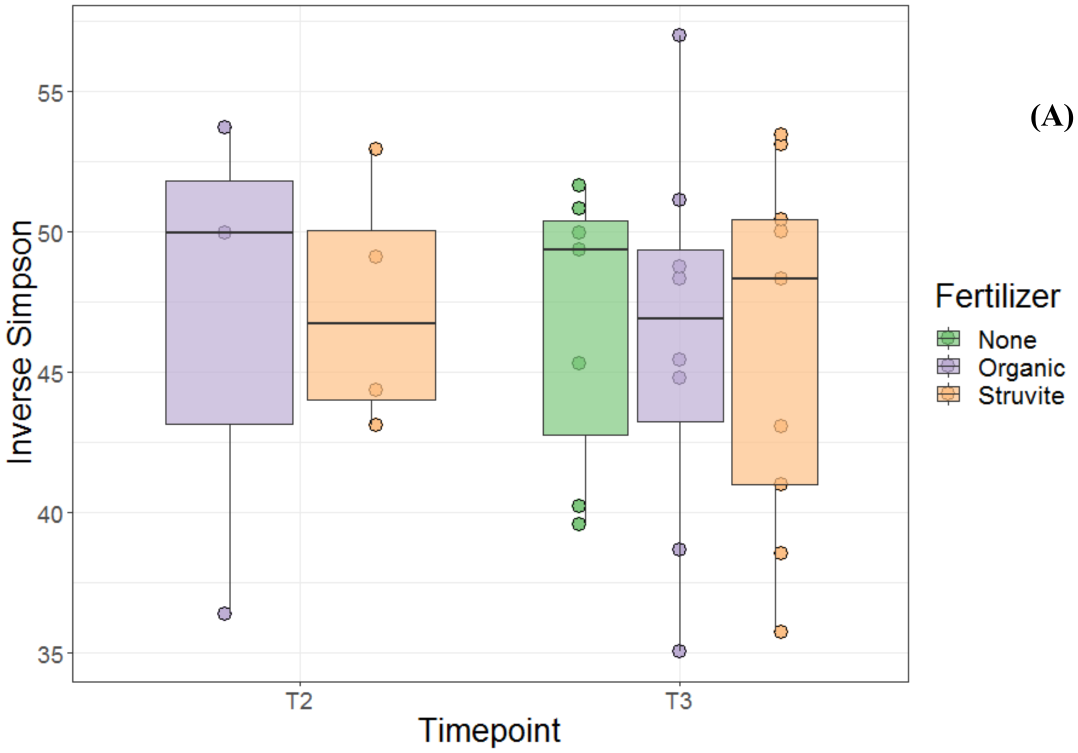

(A)

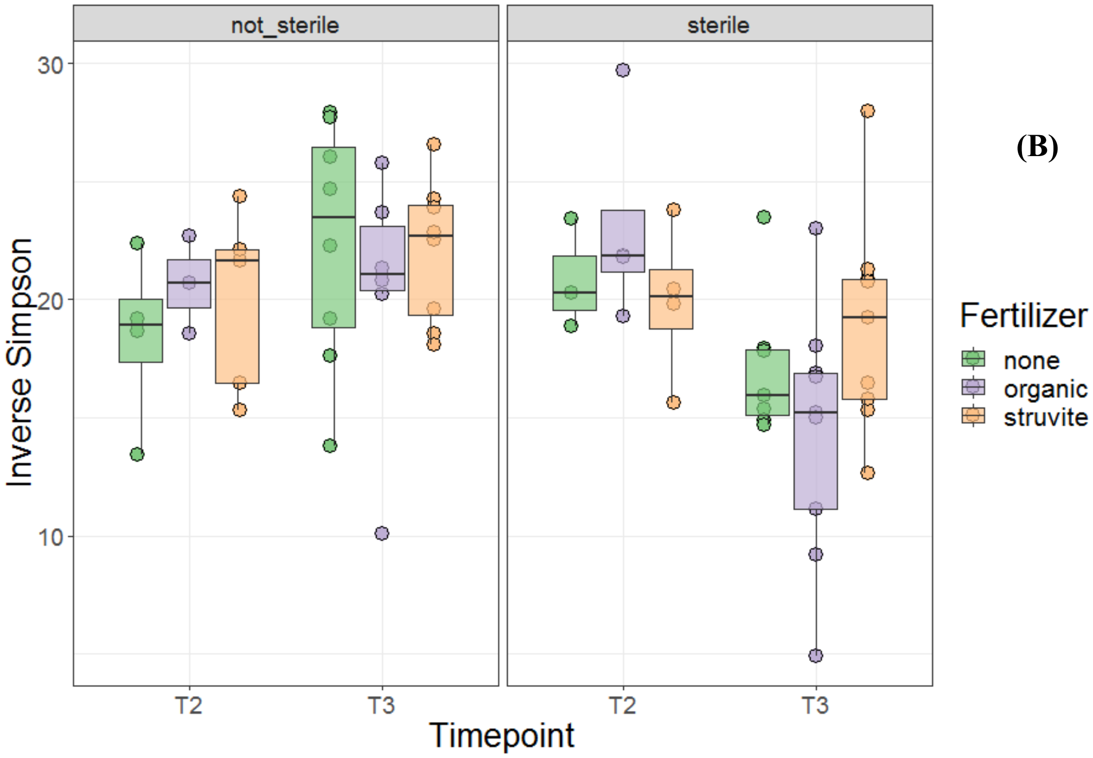

(B)

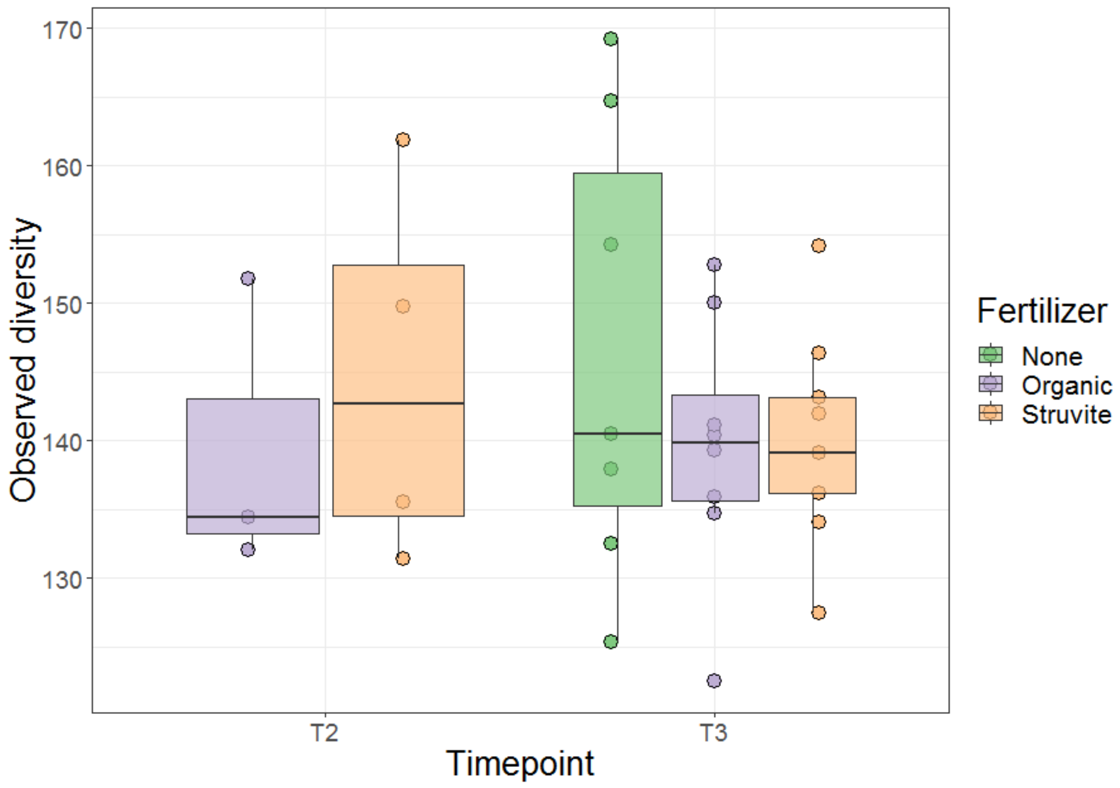
170
160
150
140
130
Observed diversity
T2
T3
Timepoint
Fertilizer
None
Organic
Struvite

(C)

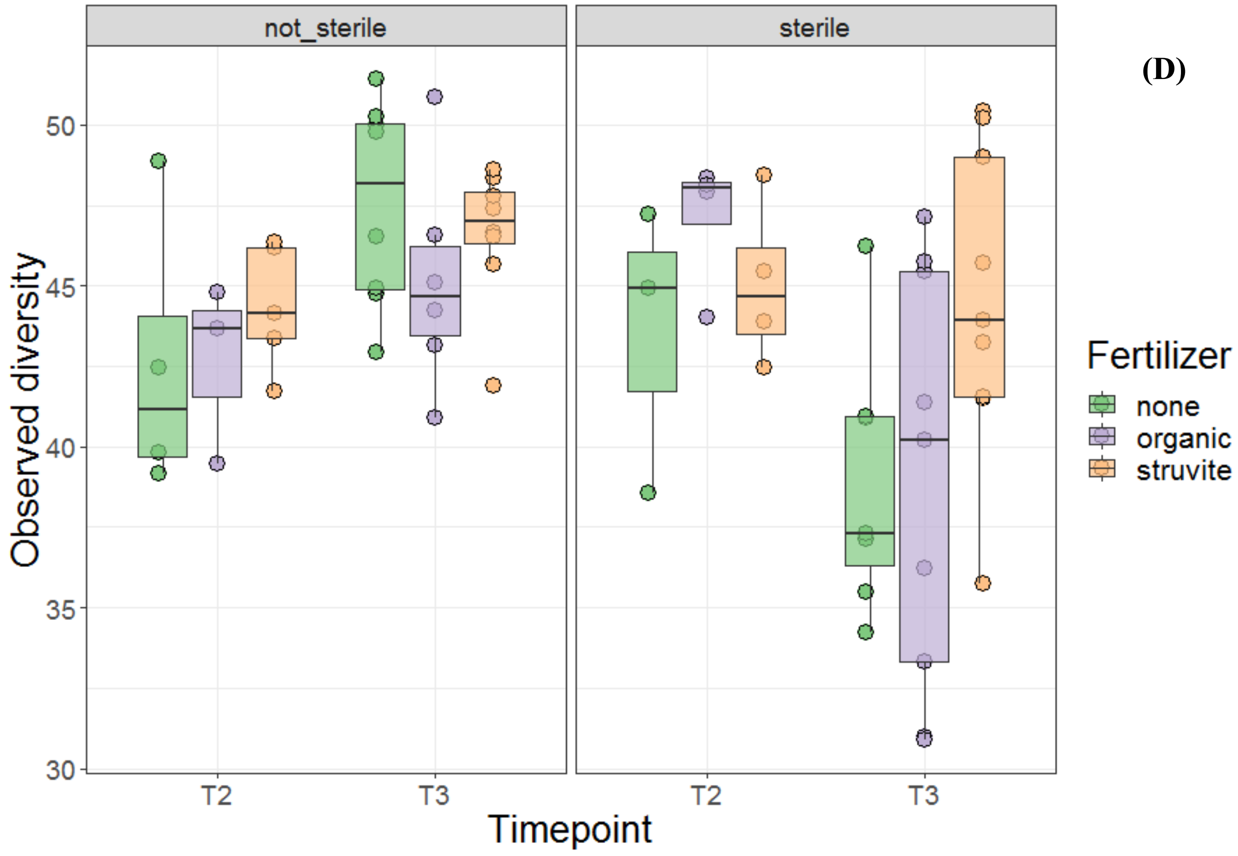
not_sterile
sterile
50
45
40
35
30
Observed diversity
T2
T3
T2
T3
Timepoint
Fertilizer
none
organic
struvite

(D)

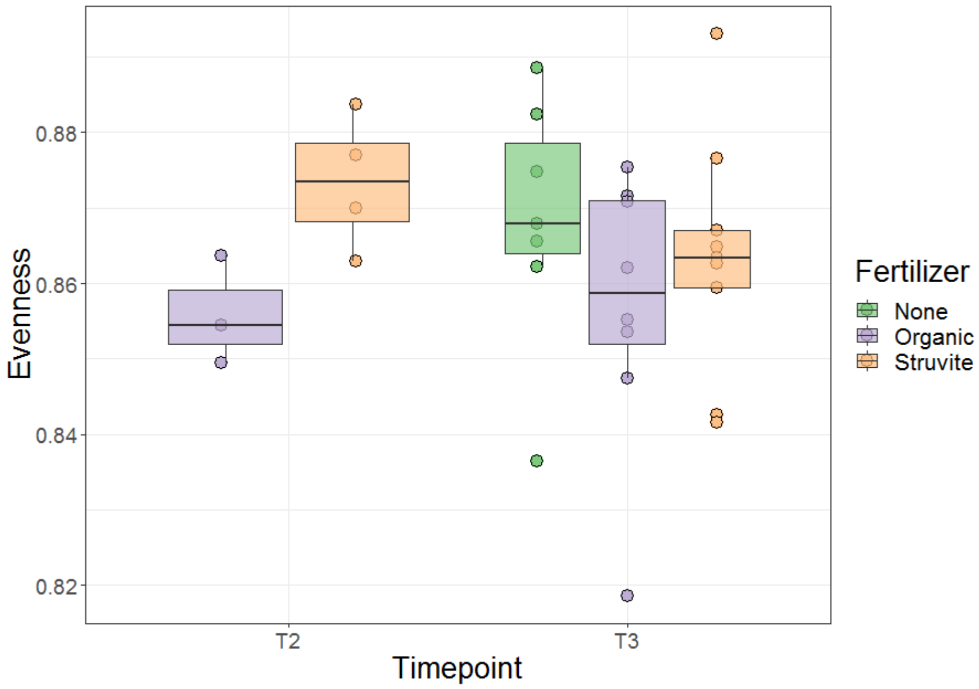

0.88
0.86
0.84
0.82
Evenness
T2
T3
Timepoint
Fertilizer
None
Organic
Struvite

(E)

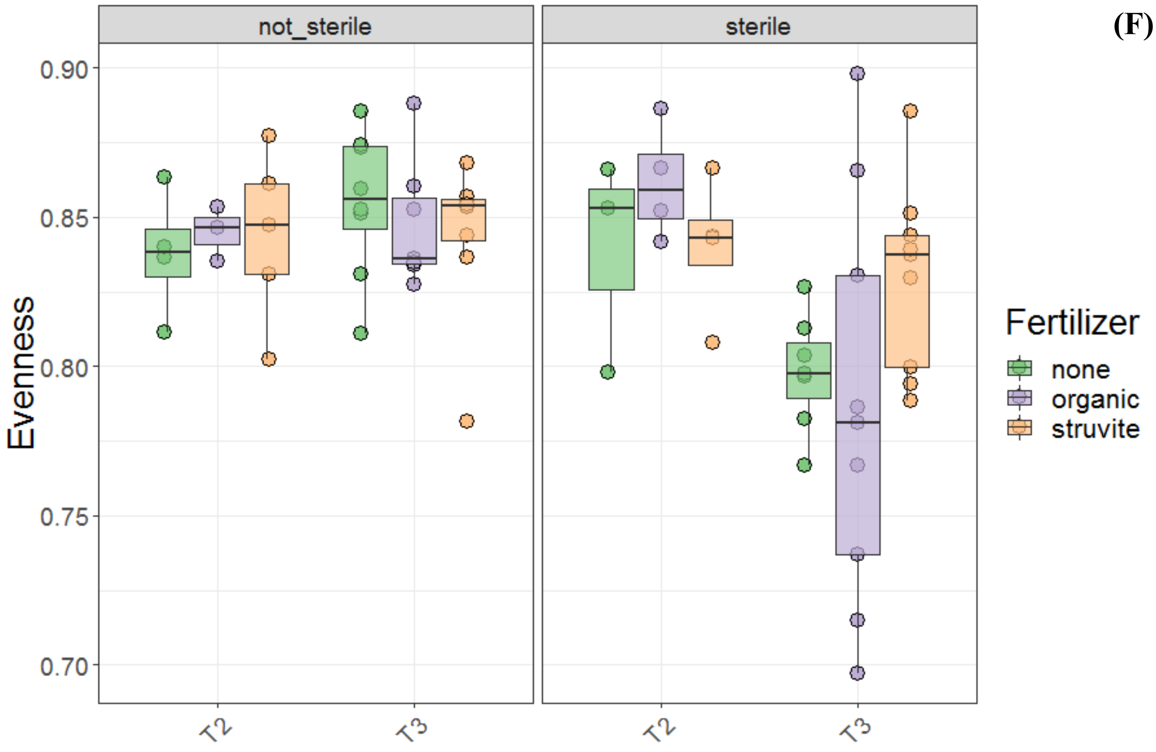

not_sterile
sterile
0.90
0.85
0.80
0.75
0.70
Evenness
T2
T3
T2
T3
Fertilizer
none
organic
struvite

(F)

Figure S3: Relative abundance of bacterial genera was similar throughout time in the rhizosheath (A) but shifted with plant presence in the rhizosphere (B)

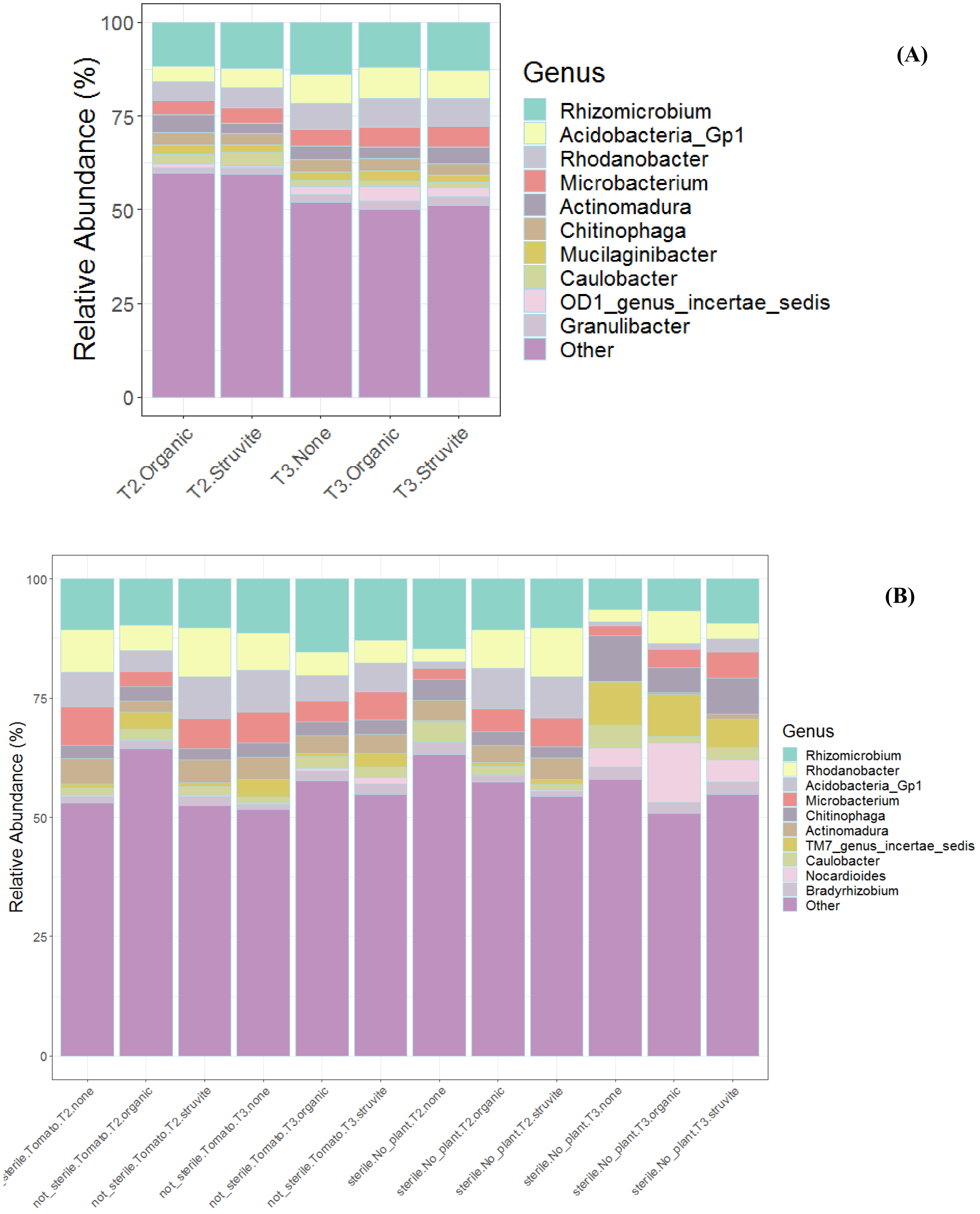

Supplement: Supplementary file 1 — Supplementary Information [file 41598_2019_45290_MOESM1_ESM.pdf]
